# MORPHIOUS: an unsupervised machine learning workflow to detect the activation of microglia and astrocytes

**DOI:** 10.1186/s12974-021-02376-9

**Published:** 2022-01-29

**Authors:** Joseph Silburt, Isabelle Aubert

**Affiliations:** 1 Biological Sciences, Hurvitz Brain Sciences Research Program, Sunnybrook Research Institute, Sunnybrook Health Sciences Centre, 2075 Bayview Avenue, Toronto, ON M4N 3M5 Canada; 2 Department of Laboratory Medicine and Pathobiology, Temerty Faculty of Medicine, University of Toronto, Toronto, ON Canada

**Keywords:** Machine learning, Microglial activation, Astrocytic activation, Cellular morphology, Focused ultrasound

## Abstract

**Background:**

In conditions of brain injury and degeneration, defining microglial and astrocytic activation using cellular markers alone remains a challenging task. We developed the MORPHIOUS software package, an unsupervised machine learning workflow which can learn the morphologies of non-activated astrocytes and microglia, and from this information, infer clusters of microglial and astrocytic activation in brain tissue.

**Methods:**

MORPHIOUS combines a one-class support vector machine with the density-based spatial clustering of applications with noise (DBSCAN) algorithm to identify clusters of microglial and astrocytic activation. Here, activation was triggered by permeabilizing the blood–brain barrier (BBB) in the mouse hippocampus using focused ultrasound (FUS). At 7 day post-treatment, MORPHIOUS was applied to evaluate microglial and astrocytic activation in histological tissue. MORPHIOUS was further evaluated on hippocampal sections of TgCRND8 mice, a model of amyloidosis that is prone to microglial and astrocytic activation.

**Results:**

MORPHIOUS defined two classes of microglia, termed focal and proximal, that are spatially adjacent to the activating stimulus. Focal and proximal microglia demonstrated activity-associated features, including increased levels of ionized calcium-binding adapter molecule 1 expression, enlarged soma size, and deramification. MORPHIOUS further identified clusters of astrocytes characterized by activity-related changes in glial fibrillary acidic protein expression and branching. To validate these classifications following FUS, co-localization with activation markers were assessed. Focal and proximal microglia co-localized with the transforming growth factor beta 1, while proximal astrocytes co-localized with Nestin. In TgCRND8 mice, microglial and astrocytic activation clusters were found to correlate with amyloid-β plaque load. Thus, by only referencing control microglial and astrocytic morphologies, MORPHIOUS identified regions of interest corresponding to microglial and astrocytic activation.

**Conclusions:**

Overall, our algorithm is a reliable and sensitive method for characterizing microglial and astrocytic activation following FUS-induced BBB permeability and in animal models of neurodegeneration.

**Supplementary Information:**

The online version contains supplementary material available at 10.1186/s12974-021-02376-9.

## Introduction

Across neurodegenerative diseases, microglia and astrocytes represent important glial cell populations that are activated in response to pathology. Depending on the context, this activation can either ameliorate or exacerbate disease progression [1, 2]. Microglial and astrocytic activation is accompanied by distinct morphological characteristics and several machine learning approaches have been developed to classify and understand activated states based on cellular morphology. Commonly, these methods deploy unsupervised learning algorithms (e.g., K-means clustering, hierarchical clustering) [3–7]. In general, these approaches aim to classify activated and non-activated cellular morphologies into distinct groups based on the similarities of their features. However, given that the activation of microglia and astrocytes exhibit a range of morphologies [4, 7–10], it remains difficult to define strict classification boundaries to accurately identify activated cells.

Supervised learning algorithms have shown promises in classifying cell types and learn rules based on patterns in labelled data to discriminate between multiple classes of features [11]. Among many applications, supervised learning algorithms have been used to identify activated microglia following traumatic brain injury [12], and to distinguish between macrophage activation states [13]. While powerful, supervised learning classifiers must be provided with labelled data, where the class label of each data point is known. For many clinical, preclinical, and basic biological applications, including for detecting activated microglia and astrocytes, standardized data sets with labelled data are not available and they are challenging to generate. Moreover, because supervised classifiers are trained using predefined classes, they suffer from an inability to discover new categories of classification, which is of interest to biologists [11].

We developed a method to identify regions of interest corresponding to activated astrocytes and microglia using a one-class support vector machine. Support vector machines in general have been widely used in biology and are both capable of modelling significant complexity while also regularizing against overfitting [14, 15]. Traditionally, support vector machines are supervised, and determine a decision boundary by evaluating the largest margin from which to separate classes of data. In contrast, one-class support vector machines require the input of a baseline class and the selection of a probability quantity (i.e., nu), which helps to define whether a datapoint should be considered consistent with the baseline, or, deemed an outlier [16]. In this way, data can be classified based on patterns learned solely from a baseline class.

Using a one-class support vector machine, we developed a novel approach to identify classes of microglial and astrocytic activation; a workflow that we termed MORPHological Identification of Outlier clUSters (MORPHIOUS). MORPHIOUS learns the feature patterns of "normal", here non-activated microglia or astrocytes, and uses this information to segment regions of cells which are classified to be "abnormal" and, therefore, inferred to be activated. This definition for activation, i.e., spatial clusters of abnormal cellular morphologies, is flexible, and thus enables the robust identification of a range of activation-associated morphologies. To facilitate its use, MORPHIOUS provides a set of ImageJ scripts to extract features from immunofluorescence images. MORPHIOUS is available to users as a stand-alone software package with a graphical user interface written in python.

To validate its utility, we used MORPHIOUS to quantify the activation of microglia and astrocytes in the hippocampus of C57BL/6 J mice treated with focused ultrasound (FUS) and intravenously injected microbubbles to induce a localized and reversible permeabilization of the blood–brain barrier, which is known to transiently activate microglia and astrocytes [17]. We further demonstrated the utility of MORPHIOUS by evaluating microglial and astrocytic activation in the TgCRND8 mouse model of amyloidosis [18]. Through our analysis, we show that MORPHIOUS can segment regions of activated microglia and astrocytes from surrounding non-activated tissue based on morphology alone.

## Methods

### Animals

For the focused ultrasound (FUS) data set, male C57BL/6 J mice (*N* = 4) at 3.5 months of age were treated with FUS unilaterally in the left hippocampus and sacrificed at 7 day (D) post-FUS. For the amyloidosis data set, 4 TgCRND8 mice [18]﻿ (2 males, 2 female) and 4 nonTg C3H/C57BL6 controls (2 males, 2 females) at 7 months of age were used. Mice were sacrificed under anesthesia of ketamine/xylazine and perfused with 4% paraformaldehyde, brains were extracted and post-fixed in 4% paraformaldehyde over night at 4 °C.

Brains were switched to 30% sucrose for > 24 h and sectioned at 40 µm using a microtome. Free floating sections were stored in cryoprotectant at -20 °C until use. All procedures were conducted in accordance with guidelines established by the Canadian Council on Animal Care and protocols approved by the Sunnybrook Research Institute Animal Care Committee.

### Magnetic resonance imaging guided focused ultrasound

Prior to FUS treatment, mice were anesthetized with 5% isoflurane, and maintained at 2% isoflurane. Fur was removed from the head using depilatory cream. A 26-guage angiocatheter was inserted into the tail vein. Animals were imaged using a 7.0-T MRI (Bruker), and T2-weighted axial scans were used to position four focal spots targeting the hippocampus. FUS was conducted using an in-house system with a spherically focused transducer (1.68-MHz frequency, 75 mm diameter, 60 mm radius of curvature) and the BBB was permeabilized using standard parameters (10 ms bursts, 1 Hz burst repetition frequency, 120-s duration) [19]﻿. At the initiation of sonication, mice were injected via the tail vein separately with Definity microbubbles (0.02 ml/kg; Lantheus Medical Imaging) and Gadovist (0.2 ml/kg, Schering AG). Each injection was followed by a 150ul flush with saline. Acoustic emissions were monitored using a polyvinylidene fluoride (PVDF) hydrophone. Acoustic pressure was increased after each pulse in a stepwise manner. Once subharmonic emissions were detected, the acoustic pressure was reduced to 25%, and maintained there for the remainder of the pulse schedule [20]. BBB permeability was confirmed based on the presence of Gadovist enhancement on T1 weighted MR images.

### Immunofluorescence staining

Serial sections (1:24) were antigen retrieved (10 mM sodium citrate, 80 °C, 30 min), washed (1X phosphate buffered saline), blocked (5% Donkey Serum with 0.3% Triton-X), and incubated for 3 days at 4 °C with primary antibodies. Primary antibodies include rabbit anti-IBA1 (Wako, cat: 016-20001; 1:500), goat anti-IBA1 (Abcam, cat: ab107159; 1:1500), goat anti-GFAP (Santacruz Biotech, cat: sc-6170; 1:250), goat anti-GFAP (Novus Biological, cat: NB100-53809; 1:2000), rabbit anti-TGFβ1 (Abcam, cat: ab215715; 1:250), rat anti-CD68 (Biolegend, cat: 137002, 1:400), goat anti-Nestin (Novus biologicals, cat: NB100-1604, 1:400), rabbit anti-S100β (Abcam, cat: ab41548; 1:1500) and mouse anti-amyloid beta (6F3D) (Dako, cat: M0872; 1:200). After washing, antibodies were incubated in secondary antibodies (Jackson Immunoresearch; 1:200) for 1 h at room temperature, washed, and mounted. For plaque staining, sections were pre-treated for 5 min in 10% formic acid, followed by 10 min in 0.1 M borate buffer (PH 8.0). After 3 days of primary antibody, sections were incubated with anti-mouse biotin (Jackson ImmunoResearch, cat: 715-065-150; 1:200) for 2 h at room temperature, washed, and incubated with streptavidin-Alexa488 (Jackson ImmunoResearch, cat: 016-540-084; 1:400) for 1 h at room temperature, washed, and mounted.

### Imaging

All images were acquired using a Zeiss Z1 Observer/Yokogawa spinning disk (Carl Zeiss) microscope. Tiled images encompassing the entire hippocampus were acquired using 40 µm z-stacks with a 1 µm step-size at with a 20X objective. All analysis was conducted using images at 20X magnification.

### Intensity and branching feature generation

All image analyses procedures were performed using Fiji/ImageJ [21]. Microglia soma, branching, and intensity measures were visualized using IBA1 immunofluorescence. Similar to previous work, astrocytes were double labelled with S100β and GFAP [22]. S100β was used to demarcate soma, while branching and intensity measures were evaluated with GFAP. For all images, a region of interest (ROI) was drawn around the hippocampus. Regions outside this ROI were cleared and therein excluded from the analysis. Z-stacked images were converted to maximum intensity projections. Prior to analysis, images were background subtracted, and despeckled. Images of astrocytes were contrast-enhanced to ensure full arborization could be detected. To collect features, for each image, a 100 µm × 100 µm sliding window was applied to the image which was iteratively translated across the image in the X and Y directions with a 50% overlap. A local threshold was first applied to the image (Method: Phansalkar, radius: 60, parameter 1: 0, parameter 2: 0). For each iteration, immunoflourescence features (Mean, IntDen, Area) were quantified using the “Measure” command, and the fractal dimension (D) was measured using the “Fractal Dimension” command. Images were further binarized (i.e., “Binarize” command) based on the local threshold, skeletonized (i.e., “Skeletonize (2D/3D)” command) and branch features were collected (“Analyze 2D/3D Features”).

### Cell soma features

Microglial and astrocytic cell bodies were segmented using custom imageJ scripts. For each 100 × 100 µm window, mean soma area, soma circularity, and nearest neighbour distance (NND), were evaluated. Soma circularity was calculated using the formula: circularity = 4π(area/perimeter^2^). For each cell soma, the nearest neighbour distance was determined as the distance between the geometric center of a cell, and the nearest neighbouring geometric cell center, as determined via the Euclidean distance.

### Segmenting microglia cell bodies

To count microglia and astrocytes, we developed a custom macro to segment and count microglia and astrocyte cell bodies. IBA1 images were first background subtracted by 50 pixels, and despeckled. Subsequently, using the MorphoLibJ library [23], we applied erosion (element: octagon, radius: 1), directional filtering (type: Max, operation: Mean, line: 6, direction: 32), morphological filter opening (element: Octagon, radius: 2), and top hat gray scale attribute filtering (attribute: Box Diagonal, minimum: 150, connectivity: 4). The image was subsequently binarized using an “IJ_IsoData” global intensity threshold. Cell body ROIs were identified using the ImageJ particle analyzer command with a size filter of 25 pixels (scale: 1.5 pixels/µm).

### Segmenting astrocyte cell bodies

S100β images were first background subtracted with a rolling ball radius of 50 pixels, and despeckled. Subsequently, using the MorphoLibJ library, we applied morphological filter opening (element: Octagon, radius: 2), gray scale attribute filter opening (attribute: Area, minimum:100, connectivity: 8), directional filtering (type: Max, operation: Mean, line: 10, direction: 32), and top hat gray scale attribute filtering (attribute: Box Diagonal, minimum: 100, connectivity: 4). A local threshold was applied to the resulting image (method: Phansalkar, radius: 60, parameter 1: -1, parameter 2: 0) which was subsequently binarized. Cell body ROIs were identified using the particle analyzer with a size filter of 30 pixels.

### Input features

Features used for identifying proximal microglia included area, mean intensity, the fractal dimension (D), number of cells, average NND, average soma size, average soma circularity, number of branches, branch length, number of branch junctions, number of triple branch points, number of branch ends, and the cellular perimeter. Features used for identifying proximal astrocytes included area, mean intensity, number of branch junctions, number of branch ends, number of slab branch pixels, number of triple points, and the cellular perimeter. Each feature was z-score normalized: *z* = (*x*_i_ - µ)/s, where *x*_i_ is individual sample value, µ is the feature mean, and s is the feature standard deviation. Both training and test-set samples were normalized based on the mean and standard deviation of the training data set. Subsequently, features were transformed using principal component analysis, and enough principal components (PCs) were selected to retain 99% of variance. This corresponded to 9 PCs for the microglia feature set and 5 PCs for the astrocyte feature set. Z-score normalization and principal component analysis were conducted using the scikit-learn module in python [24].

### Identifying proximal clusters of microglia and astrocytes

To identify outliers in hippocampal sections of FUS-treated and TgCRND8 mice, separate one-class support vector machines were trained using features from contralateral sections and control animals appropriate for each experimental group. Since outliers can represent regions with either hyperintense features, or hypointense features, the initial set of putative outliers were filtered to ensure all identified outliers had a mean intensity that was larger than a z-score of -1. These candidate outliers were subsequently spatially clustered using the density-based spatial clustering of applications with noise (DBSCAN) algorithm [25]. Spatially clustered outliers were deemed proximal clusters. Implementations for the one-class support vector machine and DBSCAN were accessed from scikit-learn [24].

MORPHIOUS requires user input for four parameters: nu, gamma, minimum cluster size, and minimum neighbour distance. The nu and gamma parameters are hyperparameters for a one-class support vector machine, and the radial-basis-function kernel, respectively. Nu reflects the percentage of normal observations which lie outside the classification decision boundary and is a regularization parameter. Gamma is a parameter for the radial basis kernel function. The minimum cluster size and distance are hyperparameters for DBSCAN which collectively defines the cluster size as the area, where the number of points greater than the minimum cluster size are within the minimum neighbour distance. By default, MORPHIOUS sets the radius to be equal to the diagonal length of the window size rounded up (142 µm). Values for nu, gamma, and minimum cluster size for each stain were optimized via a grid search (Additional file 1: Figures S2–S4).

Using the contralateral data sets, tenfold cross-validation was performed to identify the set of nu, gamma, and minimum cluster size parameters which resulted in no clustering across any control hippocampal sections. A second grid search was performed that trained on the control data set, and tested on test data set, to identify the set of hyperparameters which maximized cluster size within the test tissue (i.e., ipsilateral FUS, TgCRND8). The optimal parameters were evaluated as the set of values which maximized the clusters in the test tissue (i.e., FUS-treated, TgCRND8) while yielding no clustering in the respective control data set. Optimal parameters were identified via a separate grid search for each IBA1 (Additional file 1: Figure S2) and GFAP antibody (Additional file 1: Figure S3) in the FUS and TgCRND8 (Additional file 1: Figure S4) mice experiments.

### Identifying proximal clusters of microglia and astrocytes

**Fig. 1:**
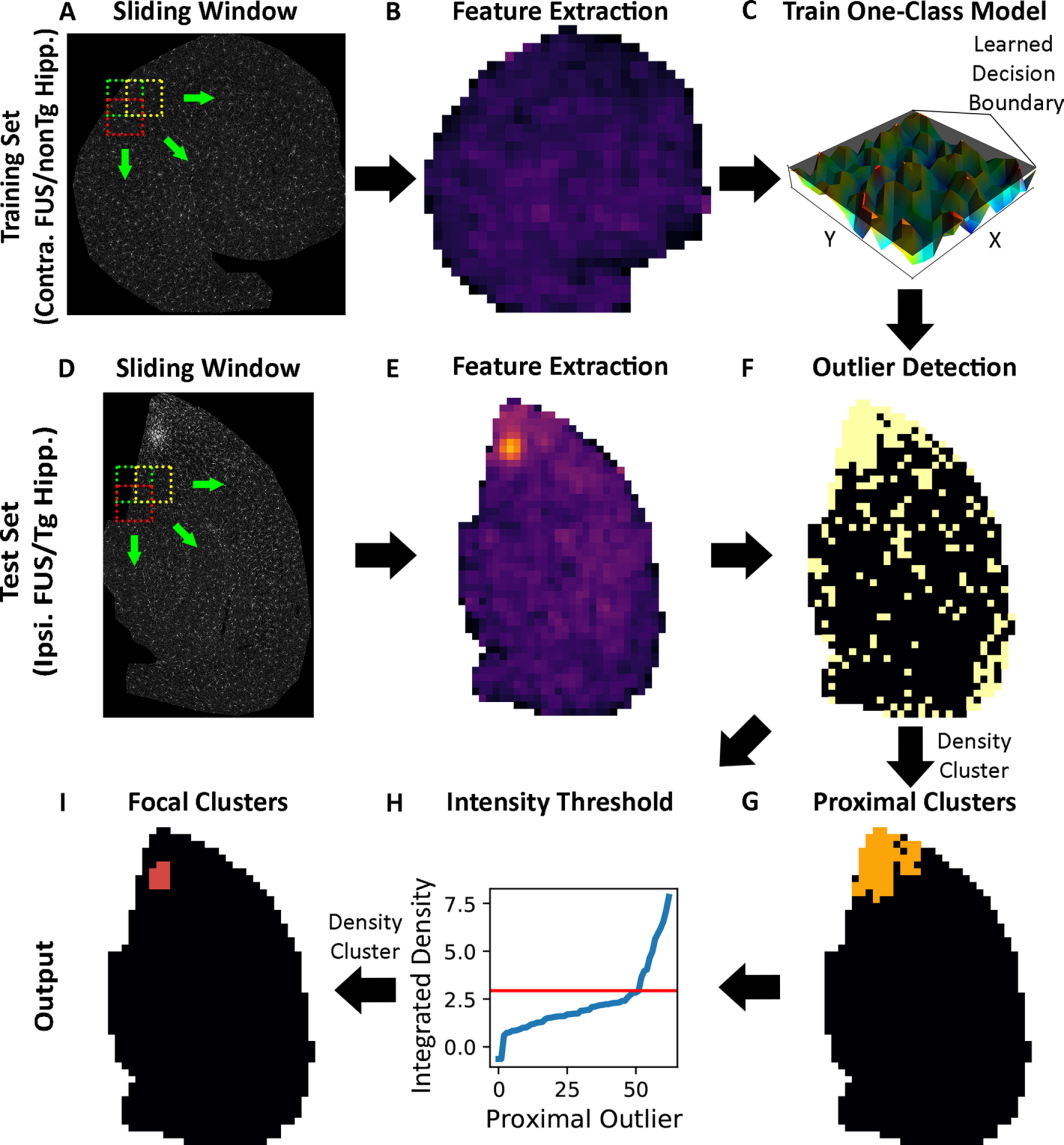
MORPHIOUS workflow trains a one-class support vector machine to identify activated glial cells. **A** sliding window is applied to control (i.e., contralateral FUS, nonTG) hippocampal sections to extract morphological features (**A**). Extracted features are used to generate a spatial feature map (**B**). Selected morphological features from control hippocampal sections are used to train a one-class support vector machine which generates a decision boundary for defining non-activated microglia and astrocytes (**C**). A sliding window is further used to extract morphological features from test-sample (i.e., ipsilateral FUS, TgCRND8) hippocampal sections (**D**, **E**). The trained model is applied to test-sample hippocampal sections to identify outlier windows (**F**). Outliers are spatially clustered using the density-based spatial clustering of applications with noise algorithm (DBSCAN) to identify proximal clusters (**G**). To identify focal clusters, the integrated density of proximal cluster windows are sorted in ascending order, and the elbow point of this curve (red line) is used as a defined threshold value (**H**). DBSCAN is applied to windows with an integrated density above the defined threshold value to establish focal clusters (**I**).Contra., contralateral; FUS, focused ultrasound; Ipsi., ipsilateral; Hipp., hippocampus; Tg, TgCRND8 mice

We further classified a second subset of microglia, termed focal microglia, which represent the most activated microglia. To identify focal microglia, first, a threshold-value was determined to identify windows of highly activated cells. Thus, for each test-set section, the IBA1 integrated density were sorted in ascending order (Fig. 1H). The elbow point of this curve corresponds to the threshold value. Proximal grid points with a mean IBA1 intensity greater than this threshold value were subsequently spatially clustered using DBSCAN, with a min cluster size of 5, and distance of 142 µm. To evaluate this elbow point, a vector was drawn to connect the first and last points (A_1_) of the integrated density curve. Subsequently, a perpendicular vector B_x_ from every datapoint in the curve was connected to A_1_. The datapoint corresponding to the largest perpendicular vector (i.e., max(|A_1_B_x_|)) was labelled as the elbow point. To ensure stability of elbow point, this procedure was iterated, and on each iteration, the first point in the curve was removed. From this procedure, the modal elbow point was used as the focal threshold value. Finally, to ensure that the integrated density IBA1 curve was sufficiently steep and reflected an exponential relationship, focal clusters were only evaluated if the magnitude of the elbow point vector (i.e., max(|A_1_B_x_|)) was greater than a threshold of 0.5, a value which worked well in our experience.

### Colocalization analysis

Pearson correlation analysis was used to assess the colocalization between IBA1 and TGFβ1, IBA1 and CD68, and GFAP and Nestin. Colocalization analysis was conducted using the coloc2 plugin in ImageJ and expressed as the Pearson correlation coefficient (R).

### Statistical analysis

In the FUS data set, differences in cellular features were analyzed using a mixed-linear model. Pairwise between-group differences in cellular features were assessed with a Sidak post-hoc test. In the TgCRND8 data set, cellular differences were analyzed using a One-Way ANOVA with a Tukey’s post-hoc test. An independent student’s *t* test was used evaluate the differences between two groups. A value of *P* < 0.05 was considered statistically significant. A linear regression was used to evaluate correlations between microglia and astrocyte cluster sizes, and between manual and automatic cell counts. All statistical analyses were conducted using SPSS (version 22, IBM).

### Source code

The MORPHIOUS source code, as well as imageJ macros, tutorials, and additional documentation are available for use at https://github.com/jsilburt/Morphious.

## Results

### Feature collection

The activation of microglia and astrocytes was induced in mice using a unilateral treatment of focused ultrasound (FUS) in the presence of microbubbles, to the left hippocampus in 14-week-old C57BL/6 J mice*.* Mice were sacrificed at 7D post-FUS, a timepoint when the activation of both microglia and astrocytes has been previously detected [26], and processed for immunohistochemical analysis. Microglia were stained with ionized calcium-binding adapter molecule (IBA1), which labels microglial processes and is upregulated with activation [27]. Astrocytes were double-stained with S100 calcium-binding protein beta (S100β) and glial fibrillary acidic protein (GFAP). GFAP, which is upregulated with astrocytic activation [2], was used to evaluate branching and intensity metrics, while S100β was used to count cells and quantify soma characteristics. Using custom ImageJ scripts, features were extracted from hippocampal slices by applying a sliding window (Fig. 1A, B). We collected features related to the fluorescence intensity, cellular surface area, branching complexity, cell location, and cell soma shape. Averaged features for each sliding window were extracted, normalized, and principal component analysis (PCA) transformed.

### Automated counting of microglia and astrocytes

To aid in feature collection, we developed two protocols using the FIJI morpholibJ package [23] to automatically count IBA1^+^ and S100β^+^ cell bodies—representing microglia and astrocytes, respectively, and measure cell soma related features. These protocols strongly correlated with manual counts (*R*^2^: 0.964 for microglia, *R*^2^: 0.959 for astrocytes) (Additional file 1: Figure S1).

### Building an unsupervised one-class classifier

**Fig. 2:**
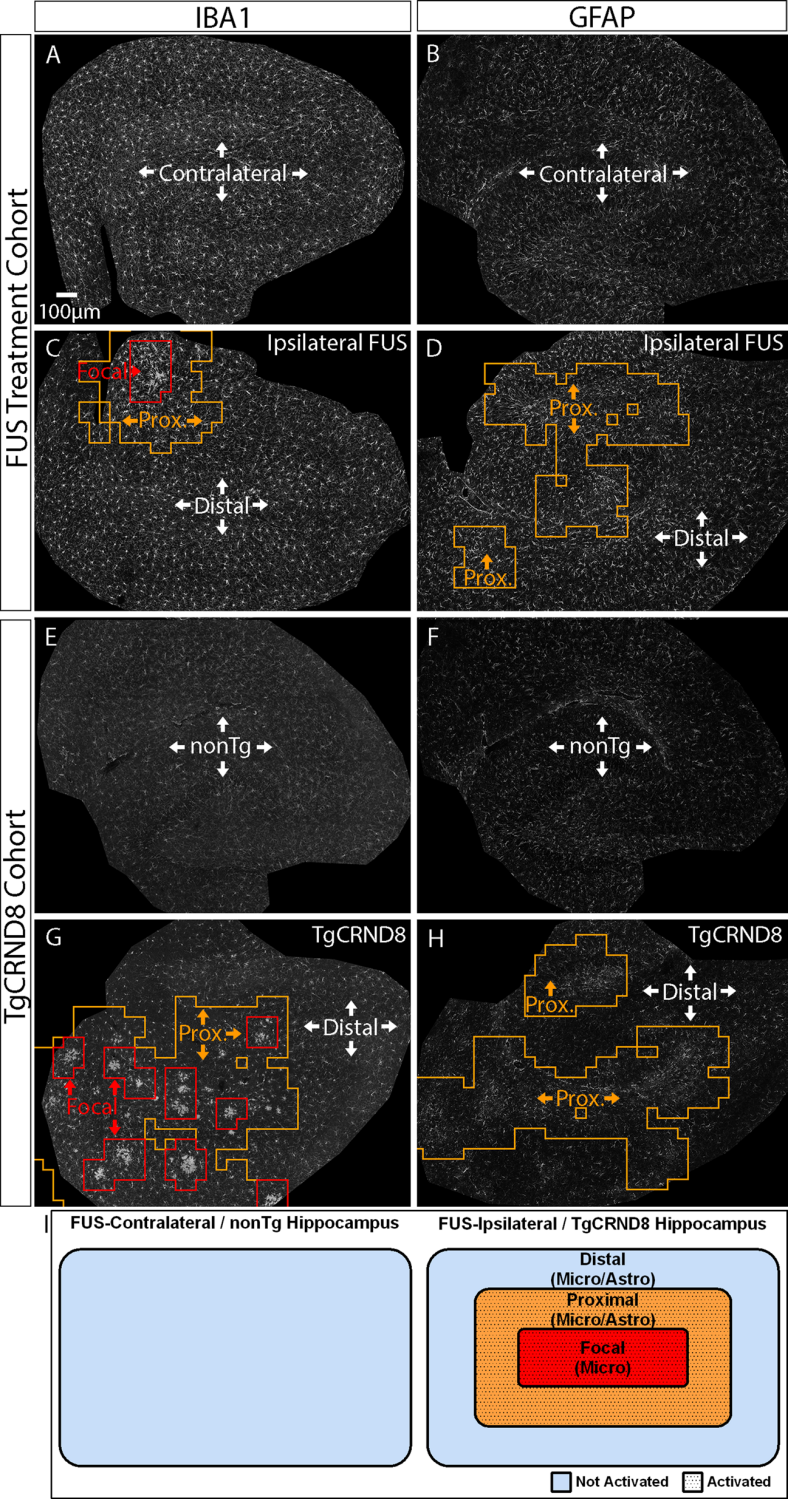
Visual demonstration of distal, proximal, and focal cluster regions identified by MORPHIOUS. Following the unilateral treatment of FUS, MORPHIOUS was trained using contralateral hippocampi stained with either IBA1 (**A**) or GFAP (**B**). When applied to the ipsilateral hippocampi stained with IBA1(**C**) or GFAP (**D**), MORPHIOUS identified proximal (orange) and focal (red) regions. Microglia and astrocytes present within the FUS–ipsilateral sections but outside proximal and focal cluster regions are referred to as distal microglia and astrocytes. Similarly, in the TgCRND8 cohort, MORPHIOUS was trained using IBA1 (**E**) or GFAP (**F**) hippocampal sections from non-transgenic (nonTg) control animals. When applied to IBA1 (**G**) or GFAP (**H**) hippocampal sections from TgCRND8 hippocampi, Focal (red) and Proximal (orange) regions of activation were identified. Microglia or astrocytes present within the TgCRND8 hippocampus but outside the identified regions of activation are referred to as distal microglia and astrocytes. (**I)** A schematic of focal, proximal, and distal cells. FUS, focused ultrasound; GFAP, glial fibrillary acidic protein; IBA1, Ionized calcium binding adaptor molecule 1; Prox., Proximal; nonTg, non-transgenic littermates of TgCRND8 mice; Tg, TgCRND8 mice

We trained a one-class support vector machine using control hippocampal sections (Fig. 1A–C). Once trained, the classifier was applied to the test-set (Fig. 1D, E). For FUS experiments, contralateral hippocampal sections were used as controls, and the FUS-treated ipsilateral sections were used in the test-set. For TgCRND8 experiments, hippocampal sections from nonTg mice were used as controls, and sections from TgCRND8 mice were evaluated in the test-set. As such, the Density-Based Spatial Clustering of Applications with Noise (DBSCAN) algorithm was used on spatial coordinates of identified outliers to generate a region of interest (ROI) corresponding to clustered activated/outlier cells (Fig. 1G). Microglia and astrocytes within these ROIs are termed proximal microglia or astrocytes, to indicate that they are located proximally to the activating stimulus. Microglia and astrocytes within the test-set tissues outside the proximal cluster regions are referred to as distal microglia and astrocytes, indicating that they are “further” from the activating stimulus than the proximal cells. This is evident from the unremarkable changes in their cellular morphologies. Among other examples, this proximal–distal terminology has been used previously to describe the spatial nature of microglial activation adjacent to an ischemic stroke [10], and to plaque pathology [17, 29]. Within proximal microglial activation clusters, we observed subareas, where microglia exhibited prominent features of activation, which we termed focal microglia. To delineate the boundary of focal clusters, DBSCAN was applied to proximal cluster outliers with an IBA1 integrated density above a threshold value (Fig. 1I). To calculate this threshold, the IBA1 integrated density for each proximal outlier window was sorted in ascending order and the elbow point of the ensuing curve was used as the threshold value (Fig. 1H, red line). A glossary of terms describing our spatial nomenclature can be found in Table 1. Moreover, representative visualizations of focal, proximal, and distal ROIregions of interest for FUS treated and TgCRND8 mice hippocampi is provided in Fig. 2.

**Table 1:** Terminology

Terms	Descriptions
Ipsilateral hippocampus	Unilateral hippocampus treated with focused ultrasound, which is subdivided by MORPHIOUS into distal, proximal, and focal regions
Contralateral hippocampus	Unilateral hippocampus not treated by focused ultrasound, which serves as control tissue for training MORPHIOUS for FUS-related experiments
TgCRND8 hippocampus	Hippocampus from TgCRND8 mice, a mouse model of amyloidosis, which is subdivided by MORPHIOUS into distal, proximal, and focal regions
NonTg hippocampus	Hippocampus from non-transgenic littermates of TgCRND8 mice, which serves as control tissue for training MORPHIOUS in TgCRND8-related experiments
Distal	Subregion within the ipsilateral FUS-treated or TgCRND8 hippocampi, where microglia and astrocytes exhibit a typical non-activated morphology
Proximal	Subregion within the ipsilateral FUS-treated or TgCRND8 hippocampi, where microglia and astrocytes exhibit an altered, activation-associated morphology
Focal	Subregion, within proximal activation clusters of microglia, corresponding to the microglia which exhibit the strongest activation-associated features

### Parameter tuning

To ensure that identified clusters represent morphologically activated cells, our learning objective was to predict no false-positive microglial or astrocytic activation clusters. Thus, we applied tenfold cross-validation across all control hippocampal sections (i.e., contralateral FUS, or nonTg) to identify hyperparameters, where no activation clusters were observed within any control hippocampal sections (Additional file 1: Figure S2, S3). Within the set of parameters which ensured no clustering among control hippocampal sections, we chose parameters which maximized the amount of activated microglia and astrocytes in the test-set.

### Classifying microglial activation following FUS

**Fig. 3:**
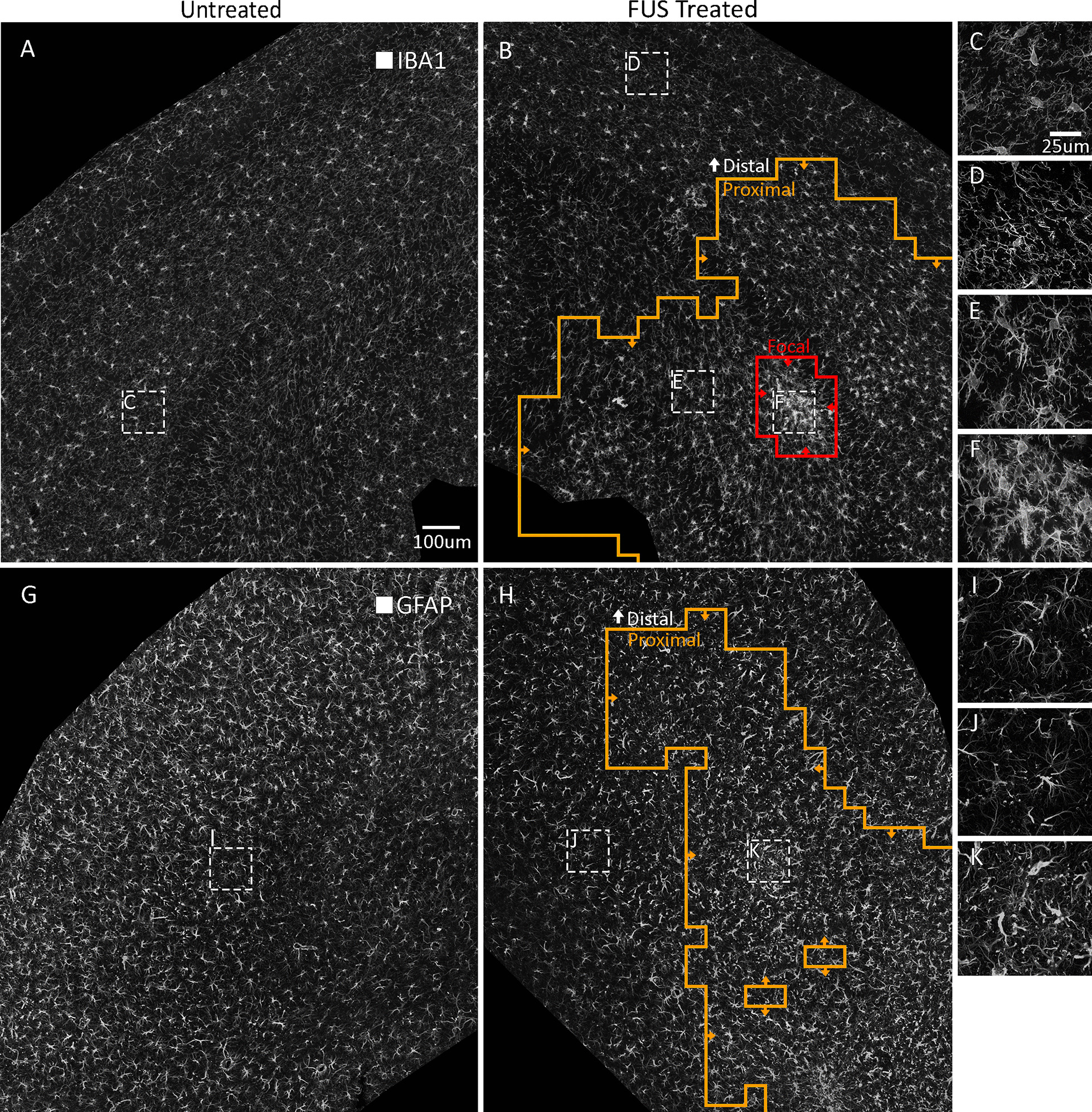
MORPHIOUS identified regions of microglial and astrocytic activation following FUS treatment. A representative staining of IBA1^+^ microglia in contralateral (**A**) and FUS-treated hippocampal sections (**B**) at 20X magnification. MORPHIOUS classified two regions of activation, proximal microglia (**B**, orange line) and focal microglia (**B**, red line). At high magnification (63X), contralateral microglia (**C**) as well as non-activated distal microglia (**D**), show a highly ramified morphology. Proximal microglia show some deramification (**E**). Focal microglia show substantial deramification and enlarged somas (**F**). A representative staining of GFAP^+^ astrocytes in contralateral (**G**) and FUS-treated hippocampal sections (**H**) is shown at 20X magnification. MORPHIOUS identified a single class of activated astrocytes, termed proximal astrocytes (**H**, orange line). At high magnification (63X), compared to contralateral (**I**) and distal (**J**) astrocytes, proximal astrocytes (**K**) show increased branching, and hypertrophy. Scale bar: 100 µm (**A**, **B**, **G**, **H**), 25 µm (**C**–**F**, **I**–**K**). GFAP, glial fibrillary acidic protein; IBA1, Ionized calcium binding adaptor molecule 1

**Fig. 4:**
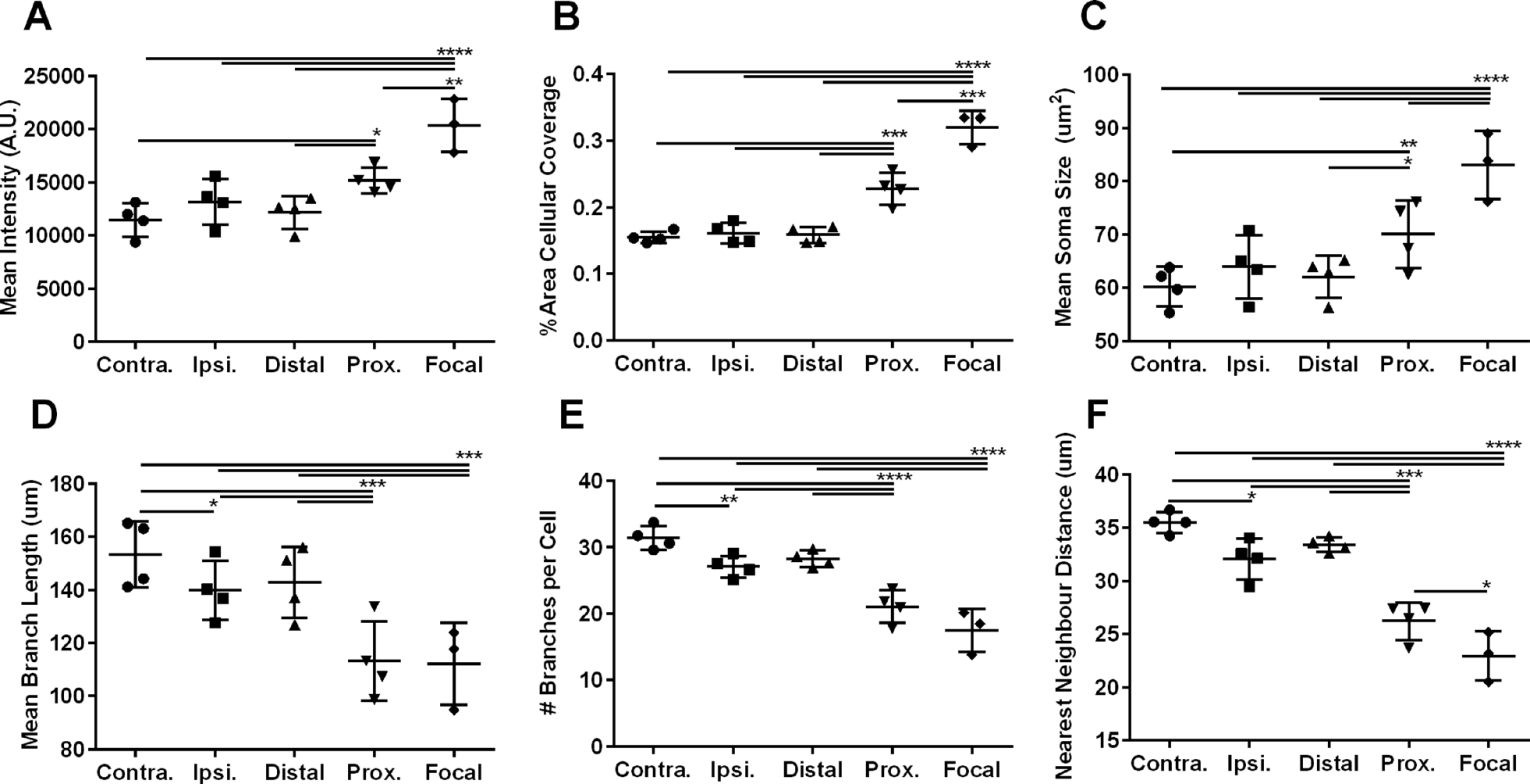
Focal and proximal microglia show morphological changes consistent with activation. The morphologies of control microglia from contralateral hippocampal sections (Contra.), ipsilateral FUS-treated hippocampal sections (Ipsi.), and MORPHIOUS classified distal, proximal (Prox.) and focal subregions within ipsilateral FUS-treated sections were compared. Cellular metrics included IBA1 mean intensity (**A**), IBA1% area coverage (**B**), mean soma size (**C**), mean branch length per cell (**D**), number of branches per cell (**E**), and the nearest neighbour distance (**F**). Groups were analyzed via a mixed linear model. Between-group differences were assessed via a Sidak’s post-hoc test. Significance: * P < 0.05; ** P < 0.01; *** P < 0.001; **** P < 0.0001. Data represent means ± SD; *N* = 4 per group (Contra., Ipsi., Distal, Prox.) and *N* = 3 per group (Focal). Contra., contralateral; Ipsi., ipsilateral; FUS, focused ultrasound; Prox., proximal

For mice treated with unilateral FUS, we trained MORPHIOUS on contralateral hippocampal sections of microglia (Fig. 3A, C) and tested it on the ipsilateral hippocampal sections (Fig. 3B, D–F). Activated microglia are known to exhibit a range of activation-associated morphologies which are characterized by progressive soma enlargement, and deramification [8, 10]. Using MORPHIOUS, we identified regions of non-activated microglia in the FUS-treated hippocampus (i.e., distal, Fig. 3D), as well as regions of proximal (Fig. 3E) and focal (Fig. 3F) microglial activation. Quantification of IBA1 immunofluorescence, soma, and branching features (Fig. 4) indicate that focal and proximal microglial activation clusters reflect distinct morphologies [8, 10]. When compared to control microglia of the contralateral hippocampus, focal microglia exhibited a 1.8-fold increased IBA1 intensity (*P* < 0.0001, Fig. 4A), a twofold increase in area (*P* < 0.0001, Fig. 4B), a 1.4-fold increase in soma size (*P* < 0.0001, Fig. 4C), a 1.4-fold reduction in branch length (*P* < 0.001, Fig. 4D), a 1.8-fold reduction in the number of branches per cell (*P* < 0.0001, Fig. 4E), and a 1.5-fold reduction in nearest neighbour distance (NND) (*P* < 0.0001, Fig. 4F). Moreover, when compared to proximal microglia, focal microglia also exhibited a 1.3-fold increase in IBA1 intensity (*P* < 0.01, Fig. 4A), a 1.4-fold increase in area (*P* < 0.001, Fig. 4B), a 1.2-fold increase in soma size (*P* < 0.0001, Fig. 4C), and a 1.2-fold reduction in NND (*P* < 0.05, Fig. 4F). Similarly, proximal microglia exhibited significant, but less pronounced change in IBA1 intensity (*P* < 0.05, Fig. 4A), area (*P* < 0.0001, Fig. 4B), soma size (*P* < 0.05, Fig. 4C), branch length (*P* < 0.001, Fig. 4D), number of branches (*P* < 0.0001, Fig. 4E), and NND (*P* < 0.001, Fig. 4F), when compared to contralateral cells.

For all assessed features, distal microglia, were statistically indistinguishable from contralateral microglia (*P* > 0.05), rendering them representative of non-activated microglia in the FUS-treated hippocampus. These findings are visualized via principal component analysis, where focal and proximal activation clusters occupy distinct regions in feature space, while distal microglia overlap with contralateral microglia (Additional file 1: Figure S5).

When conducting immunohistochemical analyses, it is important that a representative ROI is selected [30–32]. Typically this ROI should be a clearly definable area, such as a brain region (e.g., hippocampus), that is analyzed in its entirely, or through appropriate sampling [30–32]. In practice, both whole-region and sampling approaches are common [10, 26, 33–36]. To illustrate some of the advantages of MORPHIOUS, we asked whether a typical quantitative approach could detect activated microglia in our sections. Thus, we defined the analytical ROI as the entire hippocampal area and compared microglial morphologies within FUS-treated hippocampal sections to their contralateral side. Notably, ipsilateral microglia showed only small reductions in the branch length (*P* < 0.01, Fig. 4D), number of branches (*P* < 0.05, Fig. 4E), and nearest neighbour distance (*P* < 0.05, Fig. 4F), but showed no changes for other features (*P* > 0.05). These results of this whole-region analysis are in contrast with those obtained when MORPHIOUS was used to identify ROIs, where a rich set of distinct morphologies were detected (Fig. 4). Thus, by defining discrete clusters of activation, MORPHIOUS improves the sensitivity for detecting pockets of activated microglia in heterogeneous tissues when compared to a traditional analytical approach.

**Fig. 5:**
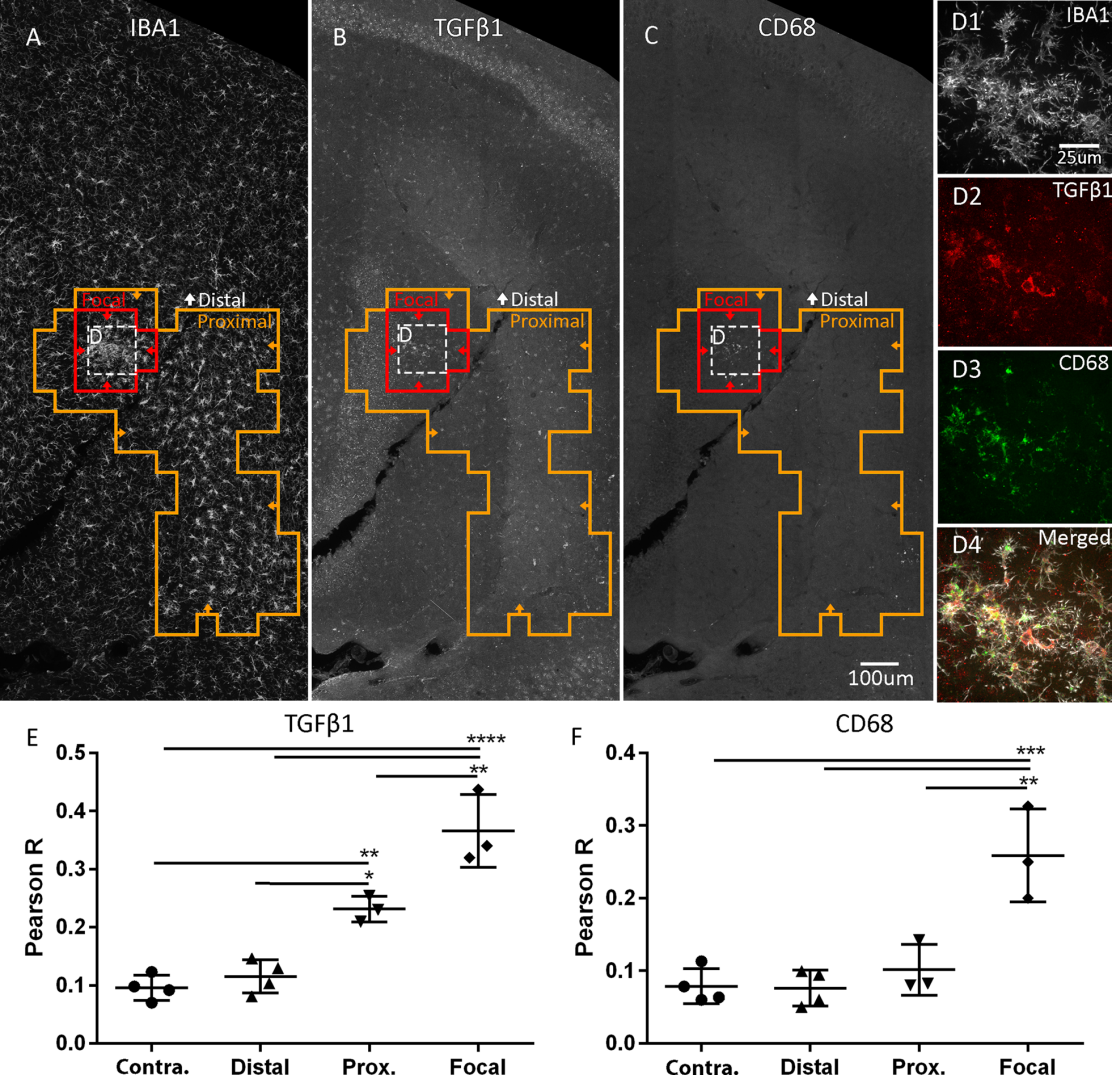
Focal and proximal microglia differentially upregulate common markers of activation. Focal (red line) microglia (**A**) colocalized with TGFβ1 (**B**, **D**), and CD68 (**C**, **D**). Proximal (orange line) microglia colocalized with TGFβ1, but not CD68. Pearson correlation was used to colocalize IBA1 with TGFβ1 (**E**) and CD68 (**F**). Images (**A**–**C**) were taken at 20× magnification. Insets (**D1**–**D4**) were taken at 63× magnification. Groups were analyzed via a mixed linear model. Scale bar: 100 µm. CD68, cluster of differentiation 68; Contra., contralateral; IBA1, ionized calcium-binding adapter molecule 1, Prox., proximal; TGFβ1, transforming growth factor beta 1,

To further validate the activated state of focal and proximal microglia, we assessed microglial activation independently by co-staining IBA1 (Fig. 5A, D1) with TGFβ1 (Fig. 5B, D2), and CD68 (Fig. 5C, D3). CD68 has traditionally been used as a marker of both pro-inflammation [37–39], and general microglial activation [40], and has been shown to be expressed by microglia following FUS [26, 41]. TGFβ1 is considered to be an anti-inflammatory microglial marker and can facilitate neuroprotection [42, 43]. Interestingly, in accordance with a gradient of activation, focal (vs. contralateral: *P* < 0.0001; vs. proximal: *P* < 0.01) and proximal microglia (vs. contralateral: *P* < 0.01) showed progressively greater colocalization with TGFβ1 (Fig. 5E). Moreover, focal, but not proximal microglia colocalized with CD68 (vs. contralateral: *P* < 0.001, Fig. 5F).

### Classifying astrocytic activation following FUS

**Fig. 6:**
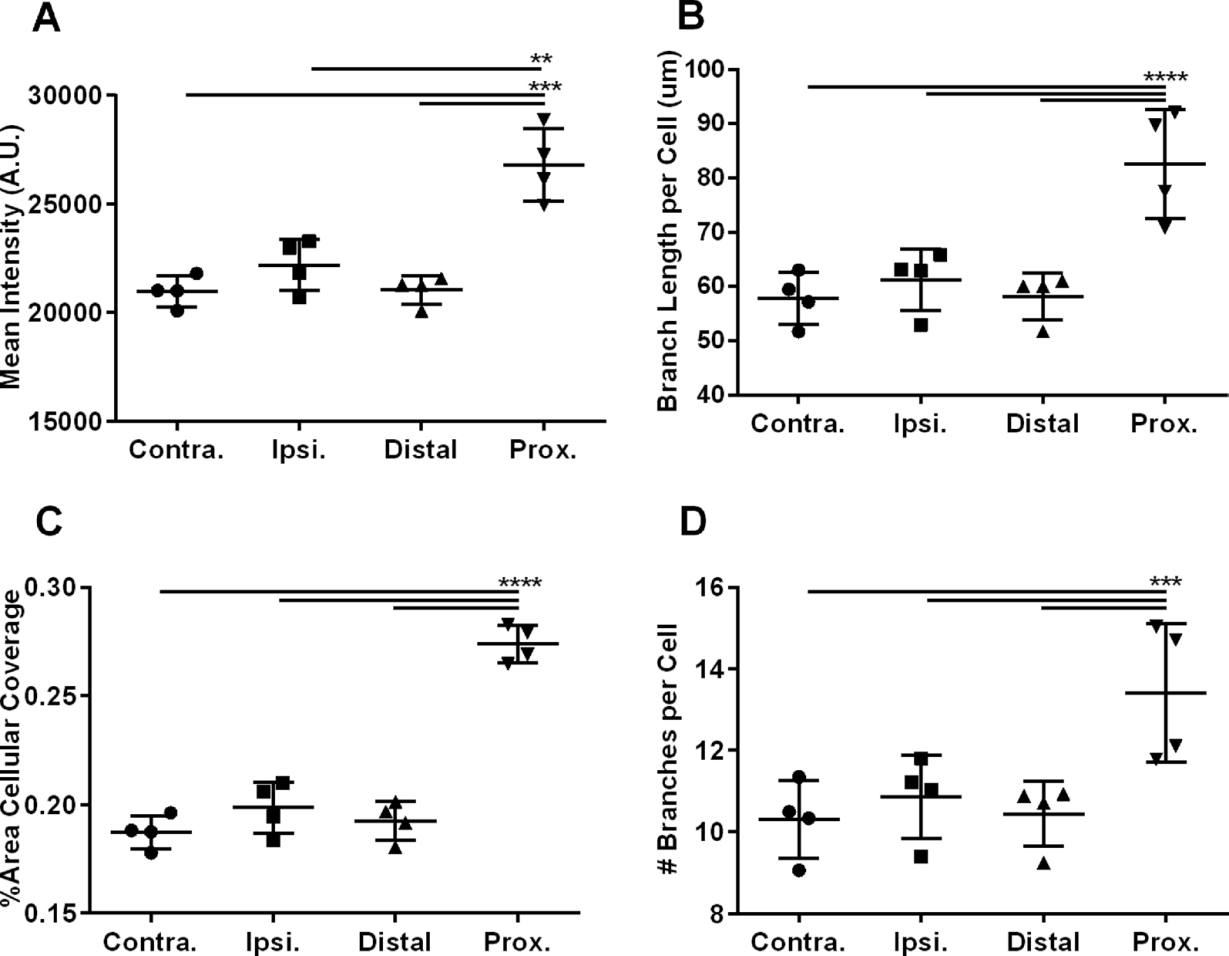
Proximal astrocytes show morphological changes consistent with activation. The morphologies of control astrocytes from contralateral hippocampal sections (Contra.), ipsilateral FUS-treated hippocampal sections (Ipsi.), and MORPHIOUS classified distal, and proximal (Prox.) subregions within ipsilateral sections were compared. Between-group differences in GFAP mean intensity (**A**), mean branch length per cell (**B**), GFAP % area coverage (**C**), and mean number of branches per cell (**D**) were assessed. Groups were analyzed with a mixed linear model, and between-group were assessed via a Sidak’s Post-hoc analysis. Significance: * P < 0.05; ** P < 0.01; *** P < 0.001; **** P < 0.0001. Data represent mean ± SD; *N* = 4 per group (Contra., Ipsi., Distal, Prox.). Contra., contralateral; FUS, focused ultrasound; GFAP, glial fibrillary acidic protein; Ipsi., ipsilateral; Prox., proximal

Next, we trained MORPHIOUS on contralateral hippocampal sections of astrocytes (Fig. 3G, I) and tested it on FUS-treated hippocampal sections of astrocytes (Fig. 3H, J, K). In response to FUS, we used MORPHIOUS to classify a single class of activated astrocytes, which we termed proximal astrocytes (Fig. 3H, K). Compared to contralateral astrocytes, proximal astrocytes exhibited a 1.3-fold increased GFAP intensity (*P* < 0.001, Fig. 6A), a 1.4-fold increased branch length (*P* < 0.0001, Fig. 6B), a 1.5-fold increased area coverage (*P* < 0.001, Fig. 6C), and a 1.3-fold increased number of branches (*P* < 0.05, Fig. 6C). As well, proximal astrocytes did not show changes to NND (*P* > 0.05, Additional file 1: Figure S6), which is consistent with in vivo findings that astrocytes do not migrate [28]. Similar to our microglial analysis, we evaluated the performance of a conventional analysis in detecting the presence of astrocytic activation following FUS in our tissue. In defining the analytical ROI as the entire hippocampal region, none of the activation-associated features of astrocytes were found to be significantly different in the ipsilateral FUS-treated side compared to the contralateral side (Fig. 6, Additional file 1: Figure S6).

**Fig. 7:**
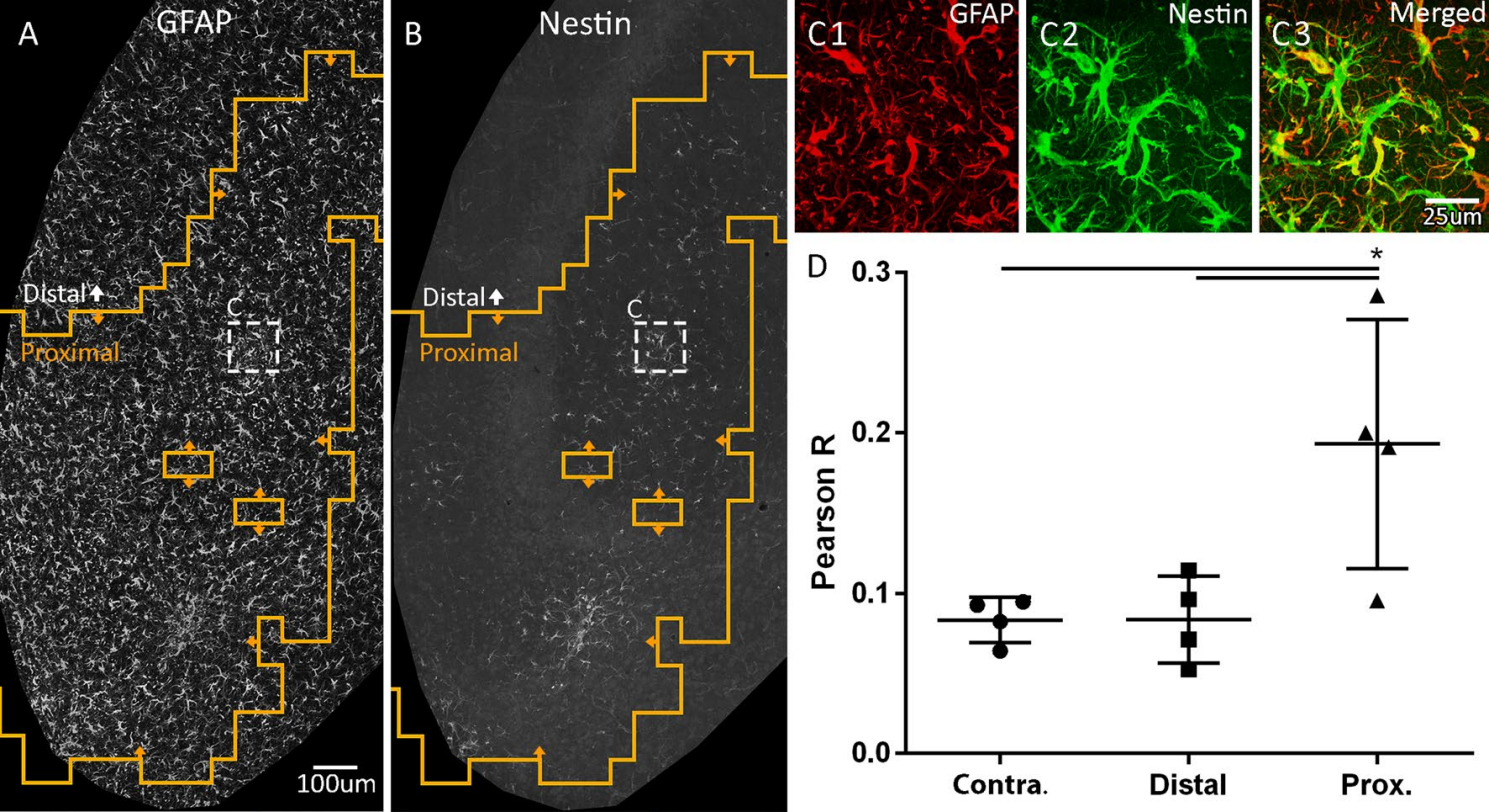
Proximal astrocytes co-express Nestin, a marker of activation. Within FUS-treated ipsilateral hippocampal sections, MORPHIOUS identified proximally activated (orange line) astrocytes (**A**) co-labelled with Nestin (**B**, **C**). Pearson correlation was used to colocalize GFAP with Nestin (**D**). Images (**A**, **B**) were taken at 20X magnification. Insets (**C1**–**C3**) were taken at 63X magnification. Groups were analyzed via a mixed linear model, and between-group differences were assessed via a Sidak’s Post-hoc analysis. Significance: * P < 0.05; ** P < 0.01; *** P < 0.001; **** P < 0.0001. Data represent means ± SD; *N* = 4 per group. Scale bar: 100 µm (**A**, **B**), 25 µm (**C1**–**C3**). Contra., contralateral; GFAP, glial fibrillary acidic protein; Prox., proximal

**Fig. 8:**
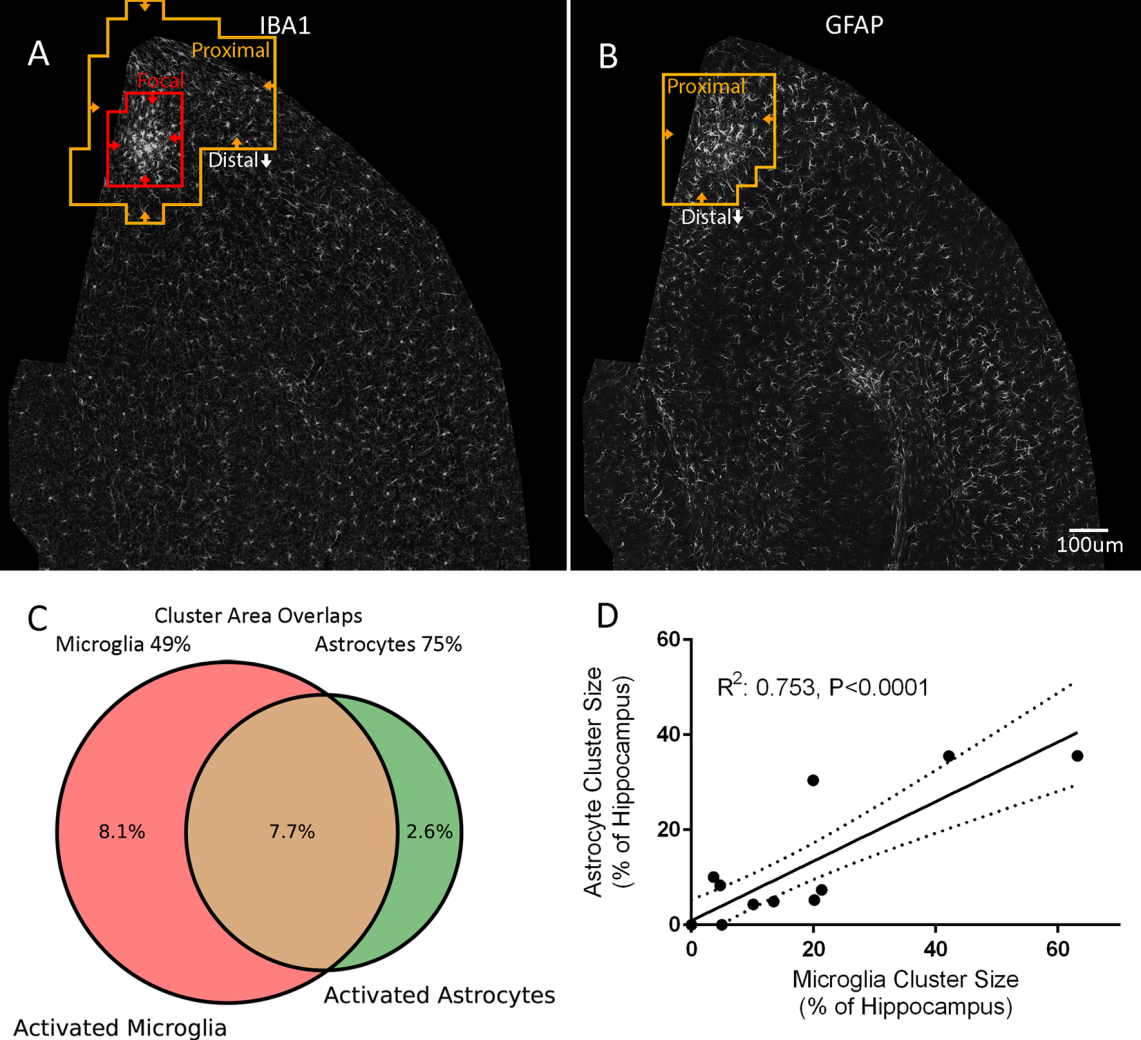
Activated microglia overlap with activated astrocytes. Proximal and focal clusters of IBA1^+^ microglia (**A**) overlap spatially with proximal clusters of GFAP^+^ astrocytes (**B**). Cluster sizes are reported as the percentage of the total hippocampal area covered by activated microglia (red) or astrocytes (green) (**C**). In total, 74.5% of astrocytic clusters overlapped with microglia clusters, while 48.7% of microglial clusters overlapped with astrocytes (brown). (**D**) Within the same section, cluster sizes for activated microglia and activated astrocytes strongly correlated. The correlation coefficient (*R*^2^) was analyzed via linear regression analysis (*N* = 16). Significance: **** P < 0.0001. Scale bar: 100 µm (**A**, **B**). GFAP, glial fibrillary acidic proteins; IBA1, ionized calcium-binding adaptor protein 1

To validate MORPHIOUS predicted clusters of astrocytic activation, we observed that proximal astrocytes colocalized with Nestin (vs. contralateral, *P* < 0.05), an intermediate filament protein which becomes upregulated during astrogliosis (Fig. 7). Moreover, there was a spatial overlap between activated astrocytes and microglia (Fig. 8A, B). In total, 15.8% and 10.3% of treated hippocampal sections were covered by activated microglia and astrocyte clusters, respectively (Fig. 8C). Of this area, 75% of activated astrocytes overlapped with activated microglia, while 49% of activated microglial clusters overlapped with activated astrocytic clusters. Moreover, proximal cluster sizes for activated astrocytes correlated with total (i.e., proximal + focal) cluster sizes for activated microglia (*R*^2^ = 0.753, *P* < 0.0001, Fig. 8D). Collectively, this suggests that both cells are responding to the common FUS stimulus, and provides additional evidence that both cell types are indeed activated.

### Classifying microglial and astrocytic activation in a mouse model of amyloidosis

**Fig. 9:**
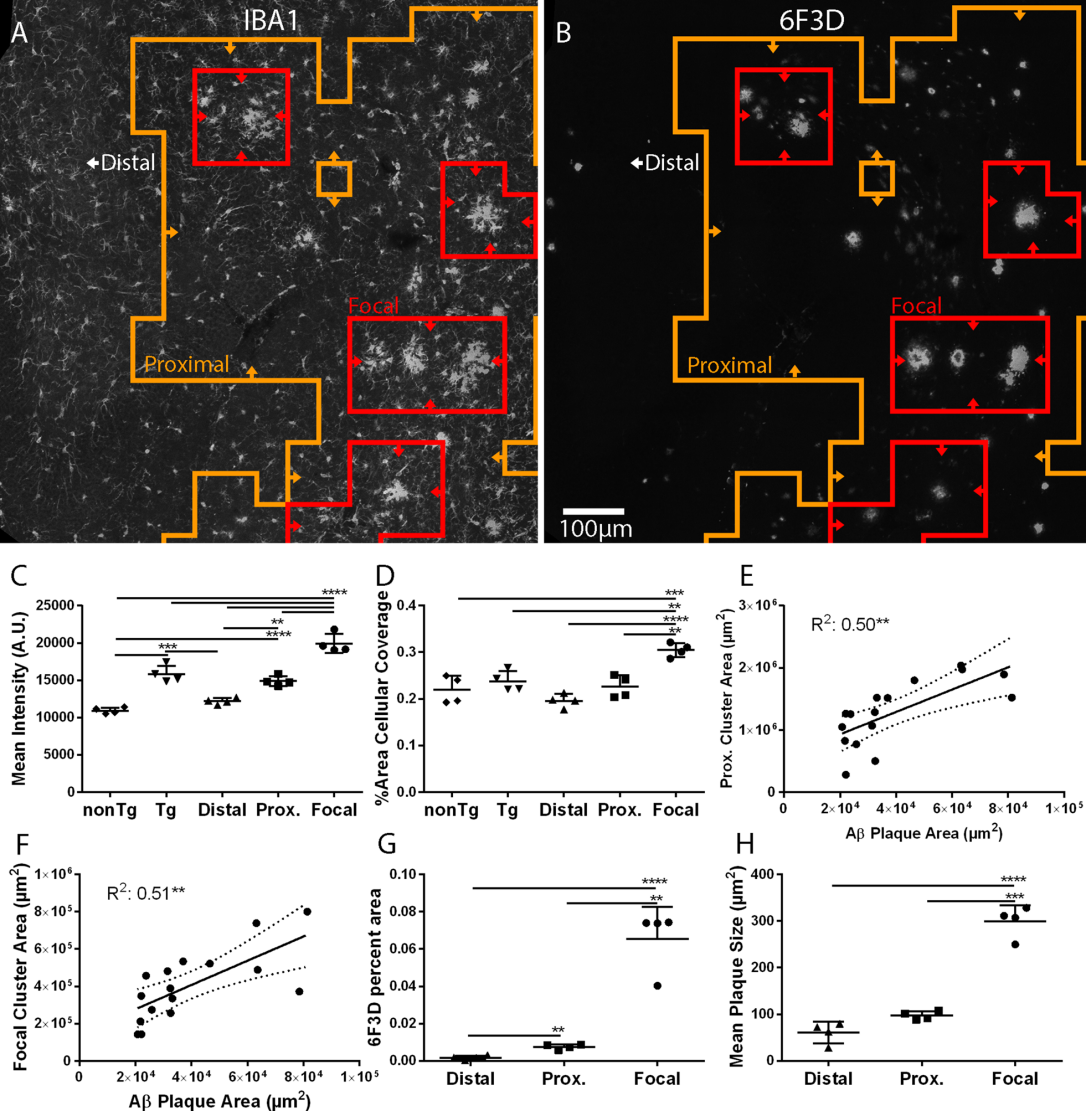
MORPHIOUS identified focal and proximal microglia in the hippocampus of TgCRND8 mice. MORPHIOUS identified focal (red line) and proximal microglia (orange line) (**A**) in association with amyloid-beta plaques (**B**). Compared with nonTg and distal microglia, proximal and focal microglia exhibited progressively higher IBA1 immunofluorescence (**C**). Focal microglia showed increased percent area coverage when compared with all other groups (**D**). Pearson correlation analysis demonstrates that proximal (**E**) and focal (**F**) microglial cluster sizes correlated with amyloid plaque load. Activated microglial clusters were associated with greater overall plaque coverage (**G**) and mean plaque size (**H**). Images (**A**, **B**) were taken at 20X magnification. Between-group differences were assessed via a one-way ANOVA with Tukey’s post-hoc analysis. Correlations were assessed via linear regression analysis and the Pearson correlation coefficient (*R*^2^) is reported. Significance: ** P < 0.01; *** P < 0.001; **** P < 0.0001. Data represent means ± SD; *N* = 4 per group. Scale bar: 100 µm (**A**, **B**). IBA1, ionized calcium-binding adapter molecule 1; nonTg, non-transgenic littermates of TgCRND8 mice; Prox., proximal; Tg, TgCRND8 mice

To assess the generalizability of MORPHIOUS to applications related to stimuli other than FUS, we evaluated microglial and astrocytic activation in 7-month-old TgCRND8 mice, a mouse model of amyloidosis. After being trained on a set of hippocampi from nonTg littermate control mice, MORPHIOUS was applied to a test-set of hippocampi from TgCRND8 mice, therein subdividing the hippocampal area into focal, proximal, and distal subregions (Fig. 9A). Focal and proximal microglia, predicted to be activated, were visually found to overlap with plaque pathology (Fig. 9B). Similar to what we observed following FUS-induced microglial activation, within TgCRND8 mice, focal microglia showed elevated IBA1 expression (Fig. 9C, *P* < 0.0001) and percent area (Fig. 9D,  *P* < 0.0001 to 0.01) when compared with contralateral, distal, and proximal microglia. IBA1 expression was also greater in proximal microglia compared to distal and nonTg microglia (Fig. 9C,  *P* < 0.0001 to 0.01).

Next, to further validate our classification predictions we asked whether microglial activation was related to plaque pathology. Both proximal (Fig. 9E, *R*^2^: 0.50, *P* < 0.01) and focal microglial (Fig. 9F, *R*^2^: 0.51, *P* < 0.01) cluster sizes correlated with amyloid plaque load. Moreover, plaque coverage was greater in both focal (Fig. 9G, *P* < 0.0001) and proximal microglial regions (Fig. 9G, *P* < 0.01) compared to distal regions. Focal microglial regions also exhibited greater plaque coverage compared to proximal microglial regions (Fig. 9G,  *P* < 0.01). Finally, compared to both distal, and proximal regions, the mean plaque size was significantly larger in focal microglial cluster regions (Fig. 9H ,* P* < 0.001 to 0.0001), indicating that focal microglial clusters are associated with larger plaques.

**Fig. 10:**
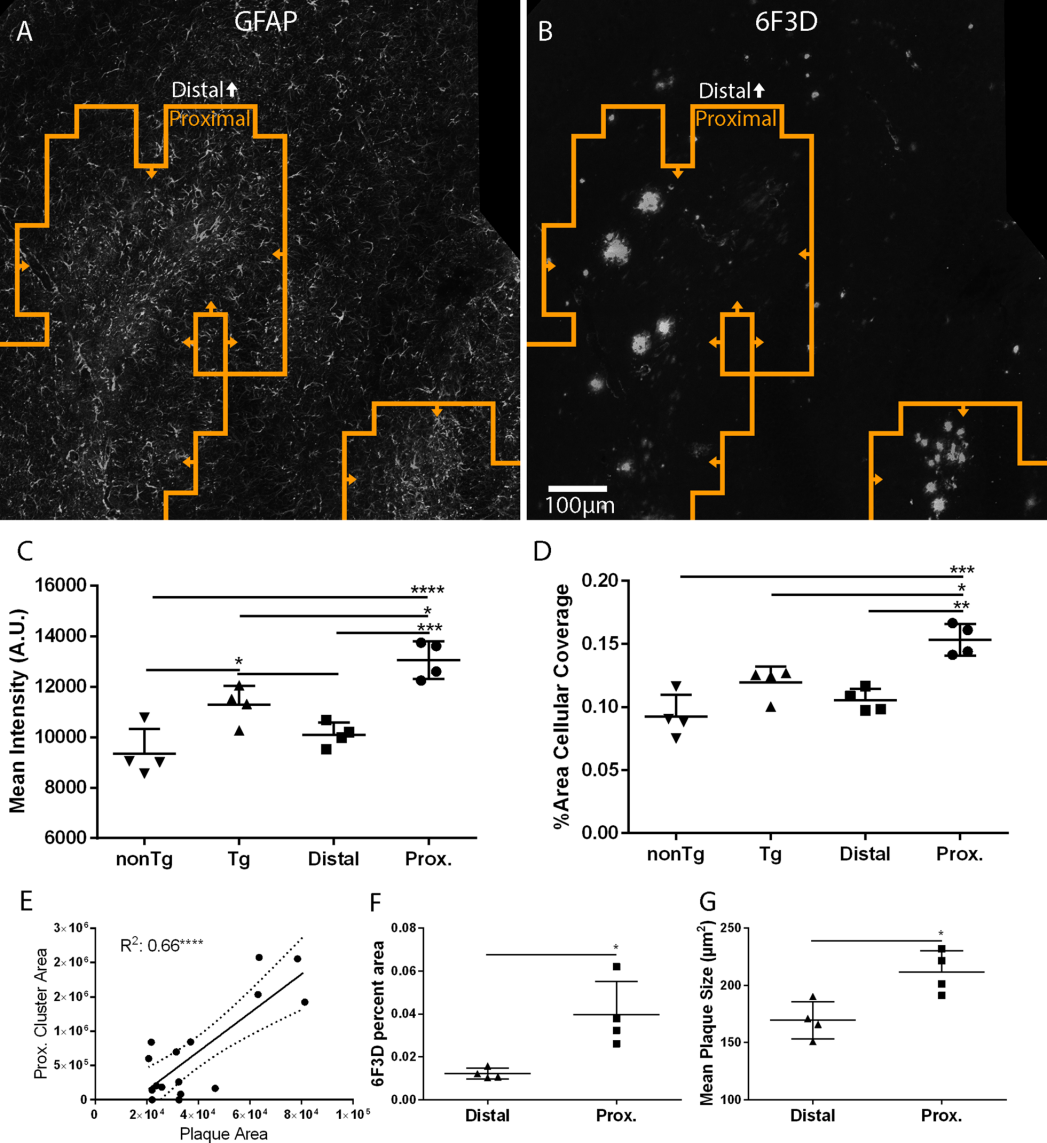
MORPHIOUS identifies proximal astrocytes in a mouse model amyloidosis. Proximal astrocytic activation clusters (orange line) (**A**) were observed in association with amyloid-beta plaques (**B**). Compared with nonTg and distal astrocytes, proximal astrocytes exhibited greater levels of GFAP immunofluorescence (**C**) and percent area coverage (**D**). Pearson correlation analysis demonstrates that proximal astrocyte cluster sizes correlated with amyloid plaque load (**E**). Proximal astrocytes exhibited increased levels of amyloid-beta plaque coverage (**F**) and plaque size (**G**). Images (**A**–**B**) were taken at 20X magnification. Between-group differences were assessed via a one-way ANOVA with Tukey’s post-hoc analysis. Correlations were assessed via linear regression analysis and the Pearson correlation coefficient (*R*^2^) is reported. Significance: * P < 0.05; ** P < 0.01; *** P < 0.001; **** P < 0.0001. Data represent means ± SD; *N* = 4 per group. Scale bar: 100 µm (**A**, **B**). GFAP, glial fibrillary acidic protein; nonTg, non-transgenic littermates of TgCRND8 mice; Prox., proximal; Tg, TgCRND8 mice

We subsequently used MORPHIOUS on hippocampal sections stained with GFAP to detect distal and proximal regions (Fig. 10A). Notably, proximal astrocytes were associated with plaque pathology (Fig. 10B). Compared with distal and nonTg astrocytes, proximal astrocyte clusters showed elevated levels of GFAP immunofluorescence (Fig. 10C * P* < 0.0001 to 0.001) and percent area coverage (Fig. 10D * P* < 0.001 to 0.01). As with activated microglia, the level of astrocytic activation correlated with plaque load (Fig. 10E, *R*^2^: 0.66, *P* < 0.0001). Compared to the distal region, the proximal astrocytic region showed greater levels of plaque coverage (*P* < 0.05), and a larger mean plaque size (*P* < 0.05). This data suggests that the detected levels of microglial and astrocytic activation by MORPHIOUS are sensitive to plaque pathology.

Collectively, the validation of classification predictions by MORPHIOUS in two animal models using different activation stimuli suggests that MORPHIOUS can be used to track pathology-associated changes in microglial and astrocytic activation.

## Discussion

In this work, we developed MORPHIOUS, an unsupervised workflow that learns a signature of "normal" microglia or astrocyte morphologies, and uses this information to generate ROIs corresponding to "abnormal" microglia or astrocytes, here referring to activated cells. The capacity to consistently identify and segment ROIs corresponding to activated microglia and astrocytes, without the need for labelled examples of activation, can improve the study of activated microglia and astrocytes in response to disease progression and following treatment. Here we demonstrated that MORPHIOUS was able to detect clusters of microglial and astrocytic activation in response to FUS-BBB modulation, and in the TgCRND8 mouse model of amyloidosis.

Activated microglia exhibit a range of morphological changes [8, 10]. Using MORPHIOUS we segmented two distinct populations: focal and proximal microglia. Consistent with activation associated morphological changes, focal microglia, and to a lesser degree proximal microglia, exhibited elevated IBA1 expression, increased soma size, decreased nearest neighbour distance and decreased branching [10]. Following FUS, focal microglia colocalized with the activation markers CD68 and TGFβ1 [42, 44], whereas proximal microglia only co-localized with TGFβ1, which itself was lower than that of focal microglia. These data support the claim that focal and proximal microglia represent two spatial subsets of microglial activation with distinct morphological and molecular identities [10, 45, 46]. Following FUS, in the same regions as activated microglia, MORPHIOUS independently identified clusters of astrocytes which were characterized by increased GFAP intensity, area coverage and branching, hallmark features of astrogliosis [2]. Moreover, proximal astrocytes colocalized with Nestin, an intermediate filament which is co-expressed with GFAP when astrocytes are activated [47]. Thus, in addition to microglia, MORPHIOUS identified regions of morphologically distinct astrocytes which exhibit features consistent with activation.

MORPHIOUS works by learning a definition of "normal" cellular morphologies from control tissues, which is subsequently used to identify spatial clusters of cells deemed to be sufficiently distinct from “normal” cells. As such, MORPHIOUS does not rigidly define the morphology of an activated microglia or astrocyte; instead, it infers a broad definition of “abnormal” activation-associated morphologies. As a result, we suggest that MORPHIOUS may be conducive towards identifying activated microglia and astrocytes in a broad range of pathologies. To support this claim, we show that MORPHIOUS could also detect focal and proximal activation clusters of microglia, and proximal activation clusters of astrocytes, in a mouse model of amyloidosis. Both microglial and astrocytic activation are known to correlate with plaque pathology [48, 49]. Similarly, we found that proximal and focal microglial and proximal astrocytic cluster sizes were responsive to amyloid burden. Collectively this suggests that MORPHIOUS can be used to detect pathological changes that are associated with microglial and astrocytic activation.

Importantly, genomic studies have clarified that the activation of microglia and astrocytes is complex and context specific [46, 50–52]. This suggests that traditional markers of activation may not be suitable for characterizing the full magnitude of microglial and astrocytic activation. MORPHIOUS provides the advantage of identifying microglial and astrocytic activation-associated morphological changes, which reduces the need for secondary activation markers. Indeed, MORPHIOUS was able to identify activated microglia in a mouse model of amyloidosis, where the molecular landscape is complex and different between microglia adjacent to plaques, phagocytosing, and those that are further away [29, 53, 54].

When quantifying cellular morphologies in immunohistochemical analyses, it is critical to choose an appropriate ROI (i.e., the denominator) by which immunological features can be normalized [30–32]. To avoid bias, it is conventional to define a ROI as brain region, or tissue type, which is either analyzed in its entirety, or, through sampling multiple fields of view [30–32]. However, quantification in this manner can be difficult in tissues with significant heterogeneity, as the presence of relatively few activated cells can be masked by the abundance of surrounding non-activated cells. This quantification problem is exemplified in the detection of small tissue perturbations as previously reported following the application of FUS. Specifically, after applying FUS to the cortex, Sinharay et al. found that despite the visual appearance of activated microglial clusters, the levels of IBA1 detected between FUS-treated and contralateral cortices were not statistically different [35]. Similarly, in our study, most features of microglial and astrocytic activation were statistically indistinguishable when comparing the entire ipsilateral FUS-treated and contralateral hippocampi (Figs. 4, 6). To increase the sensitivity of detecting morphological changes in relatively small groups of cells within a heterogenous tissue, the definition of reasonable regions of interest to focus the analysis is required (i.e., such as by a trained histologist) [30–32].

Using a data-driven approach, MORPHIOUS aims to automate this approach, and can generate discrete regions of interest of activated microglia and astrocytes. This in turn facilitates the detection and quantification of microglial and astrocytic activation not apparent through conventional analytical means. It is recognized that the identification of pathology-associated regions of interest by a trained histologist represents a gold-standard. As such, we do not claim that MORPHIOUS outperforms expert manual labelling. However, manual labelling can be labor intensive and time consuming [32]. In automatically defining ROIs, MORPHIOUS may directly aid the work of histologists in their workflows, and generate initial ROIs that can be fine-tuned as needed.

MORPHIOUS offers advantages over previous unsupervised approaches that identify activated microglia through clustering in feature space alone, such as through K-means or hierarchical clustering [3–7]. While previous methods can evaluate the putative activation of individual cells, the heterogeneity in microglia morphology poses a risk for false positives that are difficult to interpret given the nature of unlabeled data. For example, Davis et al. (2017) reported that following orbital optic nerve crush, activated microglia were found distributed among resting microglia in both the treated and untreated olfactory bulbs, a finding that merits further investigation [3]. By contrast, MORPHIOUS avoids the inclusion of individual false-positive cells by clustering through a spatial approach. While this approach prevents the identification of sparsely activated cells, or individual cells, it distinguishes MORPHIOUS from previous work by allowing it to segment whole regions of cell activation. This discrete ROI both provides an indication on the spatial extent of pathology, as well as distinguishes a region for more fine-tuned analyses. In addition, MORPHIOUS may be adaptable to applications outside of identifying microglial and astrocytic activation. Indeed, a similar one-class support vector machine approach has been used to segment the borders of tumors using MRI data [55, 56].

The lack of ground truth that could be obtained from a histologist and against which predictions could be compared, precludes the ability to report the accuracy of microglial and astrocytic classification; a limitation which is common to unsupervised quantification approaches [3, 4], including MORPHIOUS. Given that we do not have a ground truth for activated microglia and astrocytes, we cannot rule out that we are over- or under-classifying the activation of microglia and astrocytes. Tuning hyperparameters for one-class support vector machines is a critical, and often difficult task, for which a consensus on optimal methodology has yet to be reached [57]. A common technique is to maximize accuracy while minimizing the number of false-positives, based on labeled data (i.e., the class of the data is known) [55, 56]. However, examples of positive-class cases (i.e., outlier data) can be challenging to acquire. Advanced methods deploy a variety of strategies which focus on identifying patterns in the one-class itself to maximize the capacity to distinguish normal cases from outliers [57]. In our case, we leveraged two plausible biological assumptions for optimizing hyperparameters: (1) that activated microglia and astrocytes will coalesce in spatial clusters that occur in response to a stimulus. This has been well documented to occur in cases of ruptured blood vessels [10, 28], and amyloid-beta plaques [17]; and (2) that healthy control hippocampal brain tissue will not exhibit large clusters of outlier cells. Thus, in tuning our one-class support vector machine, we deployed a simple learning objective: find the set of hyperparameters which maximizes cluster size in test-set hippocampi, while ensuring that no clusters of activation are observed in control hippocampal slices.

Importantly, searching for clusters of outliers may not be suitable for images which are highly heterogenous, or, for identifying single, or small numbers of morphologically distinct cells. As with all machine learning approaches, the effectiveness of the learning model is limited by the range of features selected. To develop a simple and accessible approach, MORPHIOUS collects features through the widely used software ImageJ. It is possible that greater levels of sophistication will be required for developing features to distinguish levels of activation in microglial or astrocytic cells of higher complexity in species, such as primates, and/or following certain pathological and experimental conditions. In such case, users can input their own set of features into MORPHIOUS, such as has recently been described by other methods [4, 33], and therein expand its usability to other cases. Moreover, MORPHIOUS could be further improved with state-of-the-art convolutional neural networks that can effectively interpret features from raw images. 

## Conclusions

In conclusion, we demonstrate in two animal models that MORPHIOUS can, in an unsupervised manner, identify clusters of activated microglia and astrocytes based on morphology alone. These clusters were found to coincide with the expression of common activation markers and indicators of pathology. Quantification methods such as MORPHIOUS show promises for improving the detection of microglial and astrocytic activation in diverse contexts.

## Supplementary Information

**Additional file 1.** Additional figures.

## Data Availability

Data and materials are available on reasonable request.

## Abbreviations

CD68: Cluster of differentiation 68
DBSCAN: Density-Based Spatial Clustering of Applications with Noise
FUS: Focused ultrasound
GFAP: Glial fibrillary acidic protein
IBA1: Ionized calcium binding adaptor molecule 1
NND: Nearest neighbour distance
nonTg: Non-transgenic littermates of TgCRND8 mice
ROI: Region of interest
S100β: S100 calcium-binding protein beta
Tg: TgCRND8 mice
TGFβ1: Transforming growth factor beta 1
