# Supplementary material for: MORPHIOUS: an unsupervised machine learning workflow to detect the activation of microglia and astrocytes
Source: J Neuroinflammation. 2022 Jan 29;19:24. doi: 10.1186/s12974-021-02376-9 (PMC8800241; doi:10.1186/s12974-021-02376-9)
Supplement: Supplementary file 1 — Additional file 1. Additional figures. [file 12974_2021_2376_MOESM1_ESM.docx]

## **Additional Figures**


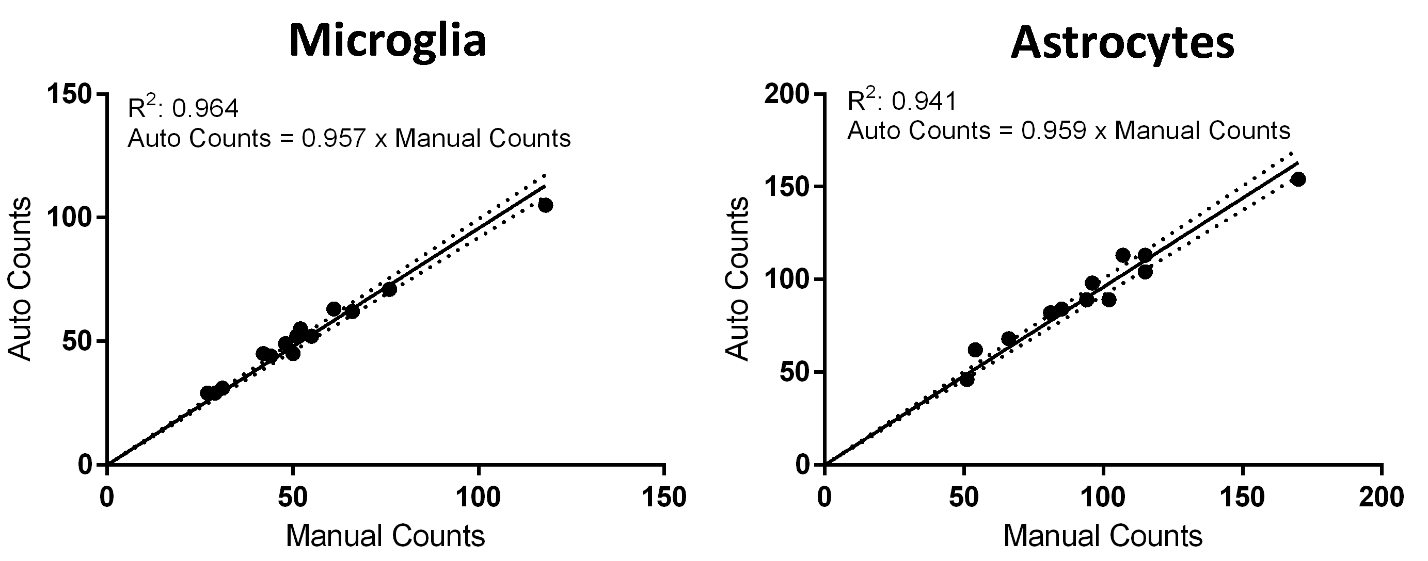


Additional file 1: Figure S1: Evaluating ImageJ macros for the automated detection of glial cell bodies. Microglia (A) and astrocyte (B) cell bodies were detected in IBA1 and S100β immunofluorescence images using macros in ImageJ. Using these protocols, predicted cell counts correlated strongly with manual cells counts, as evaluated using randomly selected fields of view (N=14, microglia, N=12, astrocytes). Comparisons were assessed using a linear regression model bounded through the origin. Significance: ****P < 0.0001.


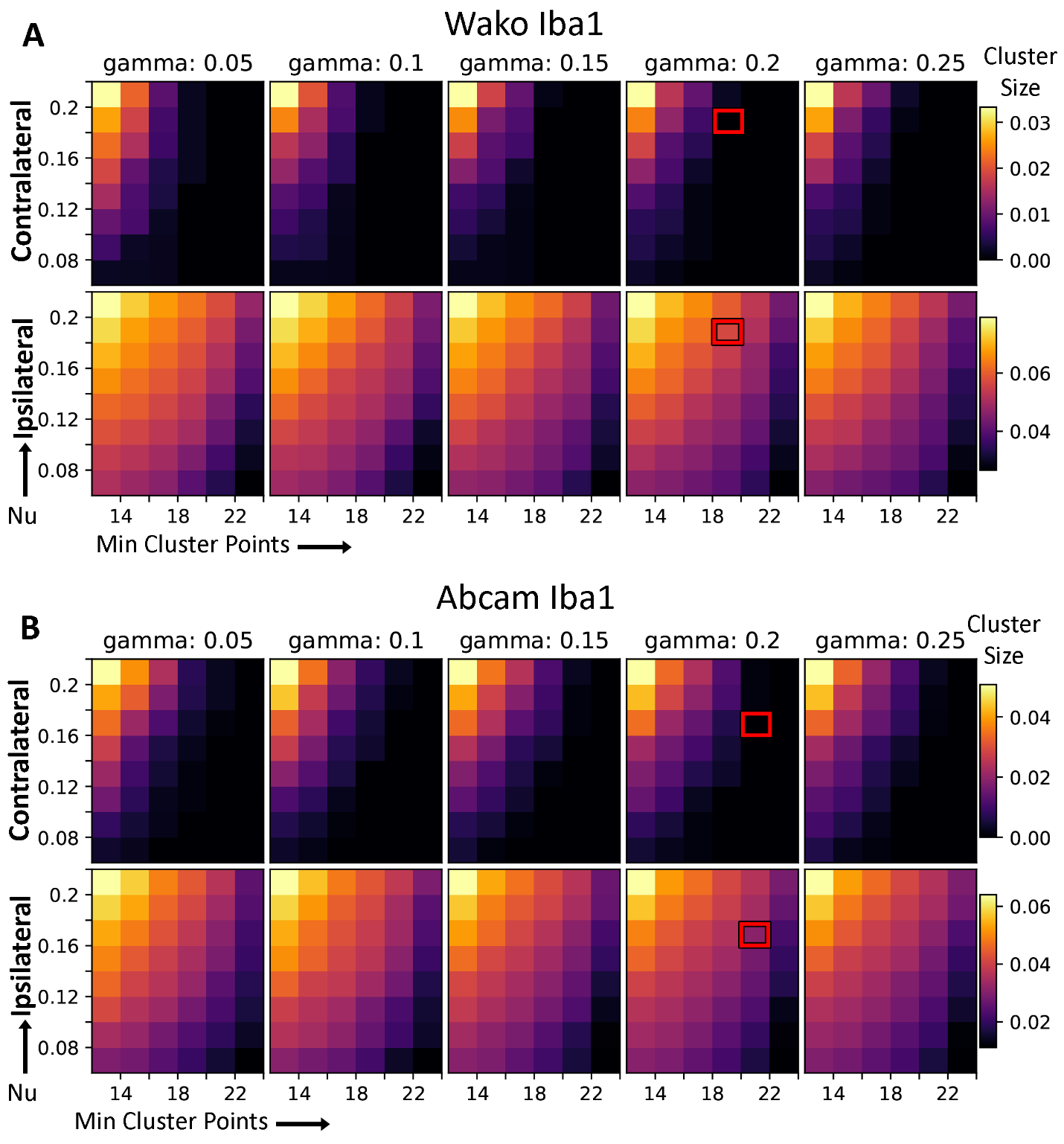


Additional file 1: Figure S2: Identifying MORPHIOUS hyperparameters for microglial activation clusters. Hyperparameters were determined for Wako (A) and Abcam (B) IBA1 antibodies. A grid search of nu, gamma, and minimum cluster points, were evaluated on the contralateral set of hippocampal sections, and subsequently the ipsilateral FUS treatment set using 10-fold cross validation. Final hyperparameters (red rectangle) were selected as the set of values which maximized the size of proximal microglia clusters in the ipsilateral FUS treatment data set, while retaining no-false positive detections in the contralateral hippocampal sections. Final parameters were nu: 0.2, gamma: 0.2, min: 20 for the Wako IBA1 dataset, and nu: 0.18, gamma: 0.2, and min: 22 for the Abcam IBA1 dataset.

Abbreviations: IBA1, Ionized calcium binding adaptor molecule 1;


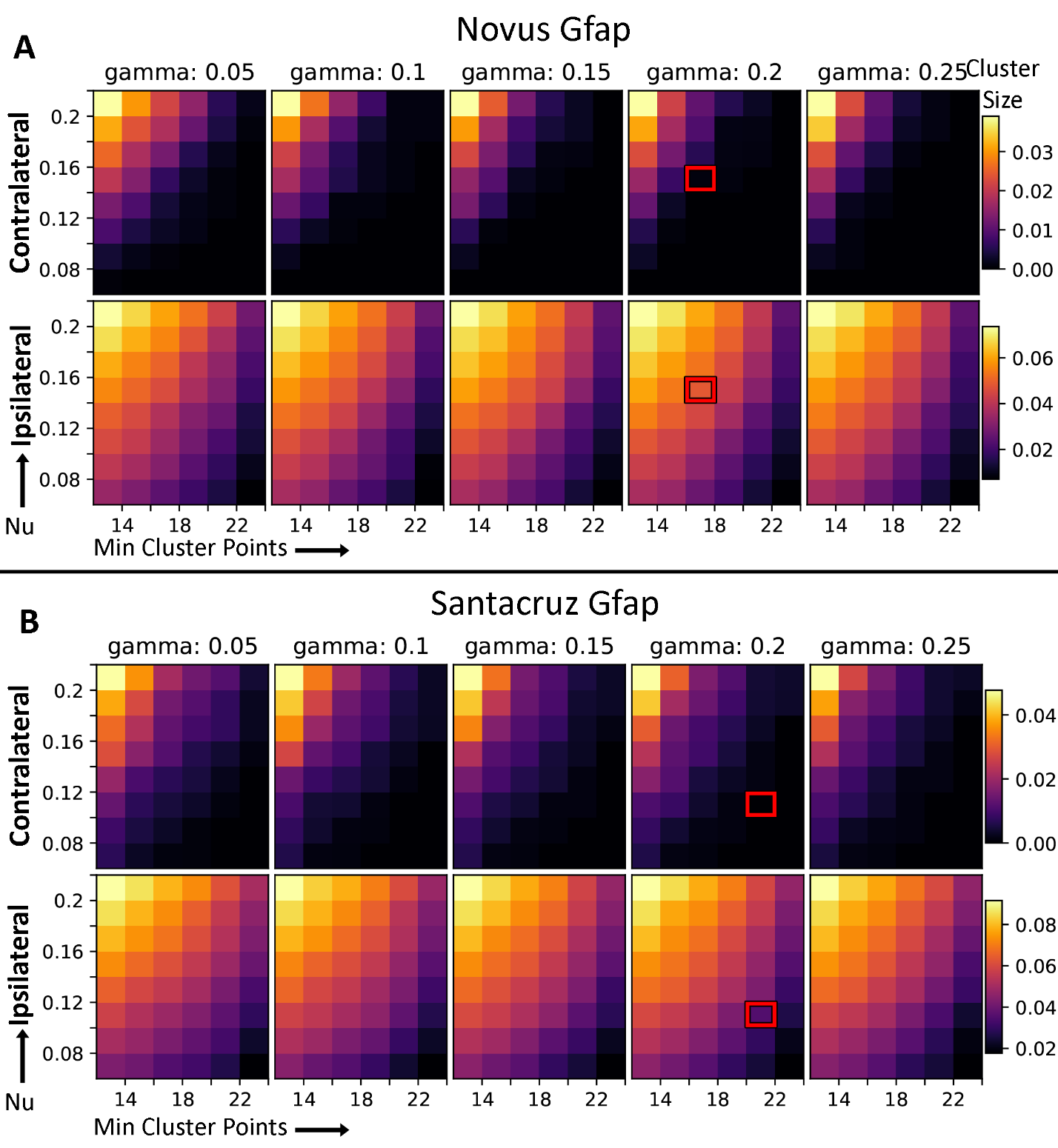


Additional file 1: Figure S3: Identifying MORPHIOUS hyperparameters for astrocytic activation clusters. Hyperparameters were determined for Novus (A) and Santacruz (B) GFAP antibodies. A grid search of nu, gamma, and minimum cluster size was evaluated on the contralateral set of hippocampal sections, and the ipsilateral FUS treatment set using 10-fold cross validation. Final hyperparameters (red rectangle) were selected as the set of values which maximized the size of proximal microglia clusters in the ipsilateral FUS treatment data set, while retaining no-false positive detections in the contralateral hippocampal sections. Final parameters were nu: 0.14, gamma: 0.2, min: 18 for the Novus GFAP dataset, and nu: 0.12, gamma: 0.2, and min: 22 for the Santacruz GFAP dataset.

Abbreviations: FUS, focused ultrasound; GFAP, glial fibrillary acidic protein


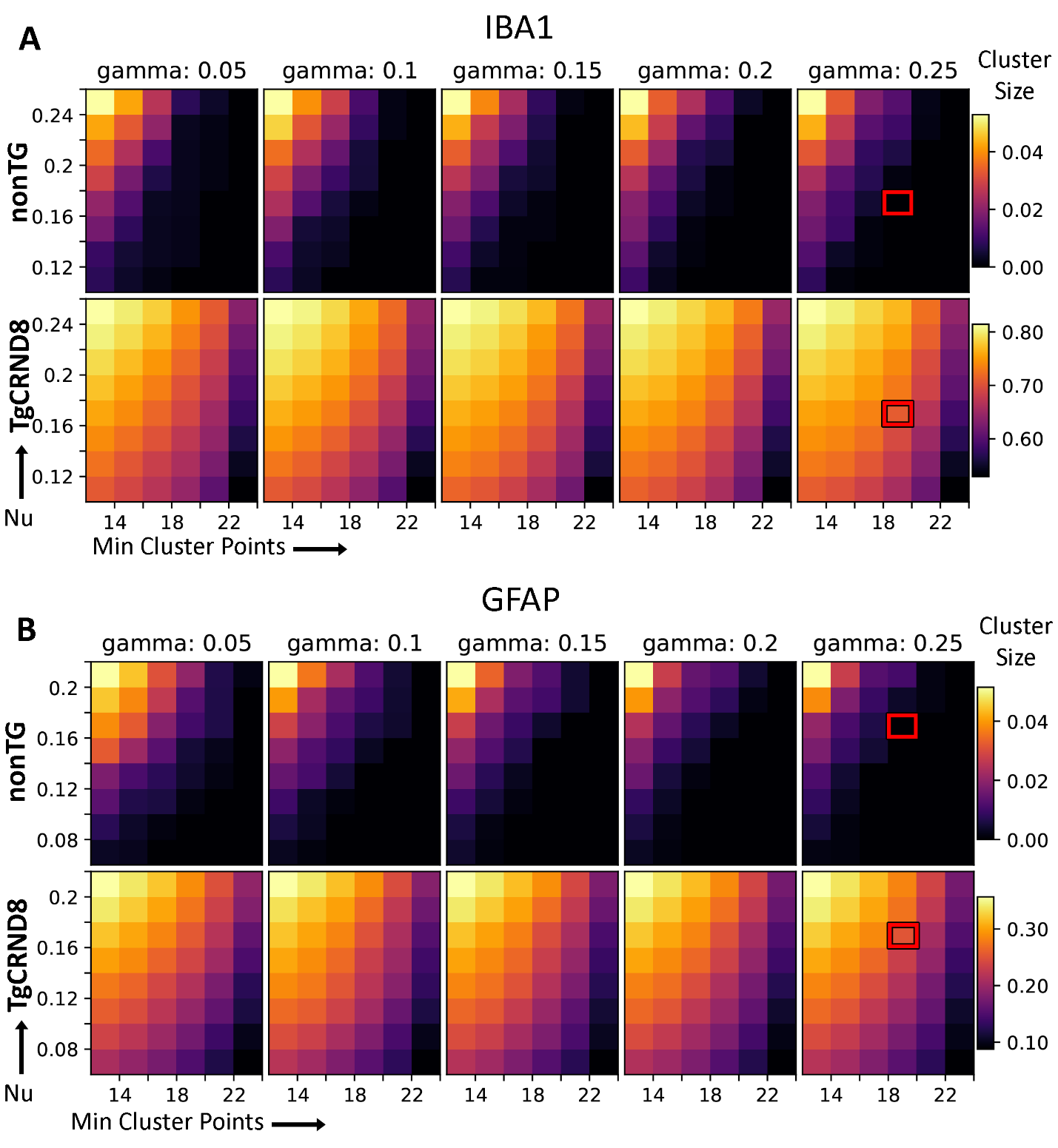


Additional file 1: Figure S4: Identifying MORPHIOUS hyperparameters for microglial and astrocytic activation clusters in the hippocampus of TgCRND8 mice. Hyperparameters were determined for IBA1 (A) and GFAP (B) antibodies. A grid search of nu, gamma, and minimum cluster size was evaluated on a control set of hippocampal sections taking from non-transgenic (nonTg) control mice, and the TgCRND8 set using 10-fold cross validation. Final hyperparameters (red rectangle) were selected as the set of values which maximized the size of proximal microglia clusters in the FUS treatment dataset, while retaining no-false positive detections in the contralateral hippocampal sections. Final parameters were nu: 0.18, gamma: 0.25, min: 20 for the IBA1, and nu: 0.18, gamma: 0.25, and min: 20 for GFAP.

Abbreviations: GFAP, glial fibrillary acidic protein; IBA1, Ionized calcium binding adaptor molecule 1; nonTG, non-transgenic littermates of TgCRND8 mice


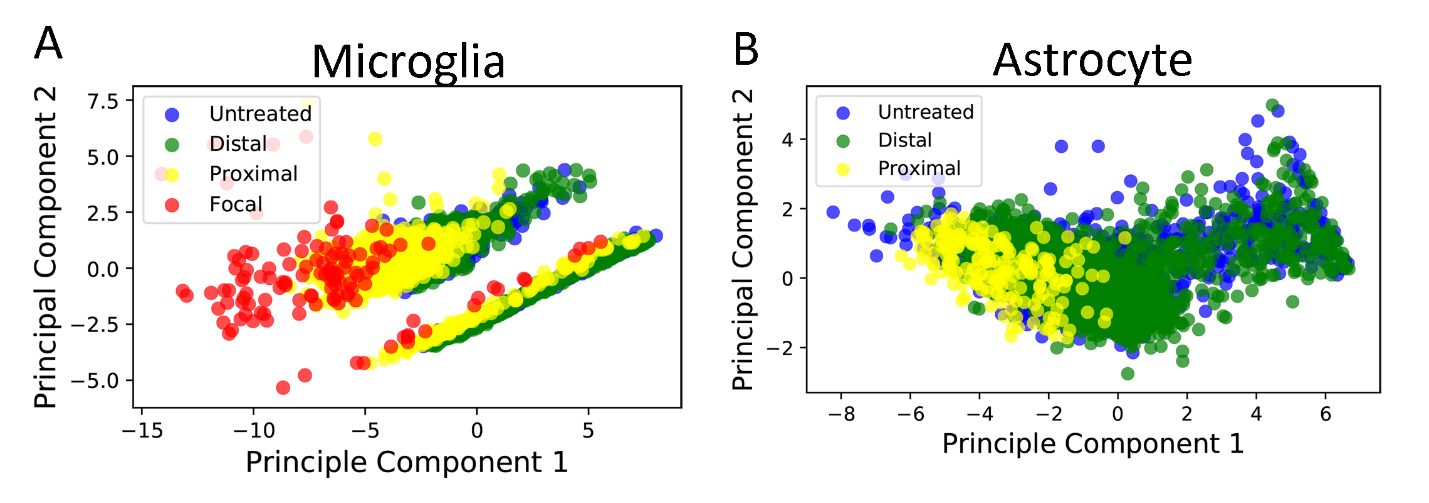


Additional file 1: Figure S5: Principal component analysis demonstrates that activated microglia and astrocytes show distinct features**.** Principle component biplots of MORPHIOUS identified activated microglia (A) and astrocytes (B) demonstrate that focal and proximal microglia, and proximal astrocytes, occupy distinct locations in feature space. Non-activated, distal microglia and astrocytes, present in ipsilateral FUS-treated hippocampal sections were indistinguishable from contralateral microglia and astrocytes, respectively. Principle component biplots are presented from representative sections for microglia and astrocytes, respectively.





Additional file 1: Figure S6: Proximally activated astrocytes do not show changes in nearest neighbour distance or soma size. The morphologies of astrocytes from contralateral hippocampal sections (Contra.), ipsilateral FUS-treated hippocampal sections (Ipsi.), and MORPHIOUS classified distal, and proximal (Prox.) regions were compared. Astrocyte somas were segmented using the soma marker S100β, via custom ImageJ scripts. Differences in soma size (A) and nearest neighbour distance (B) were analyzed via a mixed linear model and pairwise between-group differences were assessed via a Sidak’s post-hoc analysis. Data represent means ± SD; N = 4 per group (Contra., Ipsi., Distal, Prox.).

Abbreviations: Contra., contralateral, Ipsi., ipsilateral; Prox., proximal;
